# Ranking pre-trained speech embeddings in Parkinson's disease detection: Does Wav2Vec 2.0 outperform its 1.0 version across speech modes and languages?

**DOI:** 10.1016/j.csbj.2025.06.022

**Published:** 2025-06-07

**Authors:** Ondrej Klempir, Adela Skryjova, Ales Tichopad, Radim Krupicka

**Affiliations:** a Department of Biomedical Informatics, Faculty of Biomedical Engineering, Czech Technical University in Prague, Sitna Square 3105, Kladno, Czech Republic; b Department of Biomedical Technology, Faculty of Biomedical Engineering, Czech Technical University in Prague, Sitna Square 3105, Kladno, Czech Republic

**Keywords:** Wav2vec 1.0, Wav2vec 2.0, Parkinson's disease, Speech modes, Classification

## Abstract

Speech and language technologies are effective tools for identifying the distinct speech changes associated with Parkinson's disease (PD), enabling earlier and more accurate diagnosis. Models leveraging recent advancements in self-supervised speech pretraining, such as Wav2Vec, have demonstrated superior performance over traditional feature extraction methods. While Wav2Vec 2.0 has been successfully utilized for PD detection, a rigorous quantitative comparison with Wav2Vec 1.0 is needed to comprehensively evaluate its advantages, limitations, and applicability across different speech modes in PD. This study presents a systematic comparison of Wav2Vec 1.0 and Wav2Vec 2.0 embeddings across three multilingual datasets using various classification approaches to classify normal (healthy controls; HC) and PD-affected speech. Additionally, both Wav2Vec 1.0 and 2.0 were benchmarked against traditional baseline features across diverse linguistic contexts, including spontaneous speech, non-spontaneous speech, and isolated vowels. A multicriteria TOPSIS approach was employed to rank feature extraction methods, revealing that Wav2Vec 2.0 excelled across speech modes, with its first transformer layer demonstrating the best performance for classifying read text and monologue, and its feature extractor performing best in vowel-based classification. In contrast, Wav2Vec 1.0, while generally outperformed by Wav2Vec 2.0, still provided a more efficient alternative with competitive performance. Finally, we combined selected layers from both architectures and have demonstrated improved diagnostic accuracy in vowel-based classification. This comparative analysis underscores the strengths of both Wav2Vec architectures and informs their optimal use in PD detection.

## Highlights

• Systematic comparison of Wav2Vec 1.0 and 2.0 on three multilingual datasets.
• Evaluation of advantages, limitations, and use across different speech modes.
• A multicriteria TOPSIS approach was used to rank feature extraction methods.
• V2 outperformed V1 in most experiments conducted.
• Wav2Vec 1.0 is a fast, competitive method for Parkinson’s disease detection.

## 1 Introduction

The integration of artificial intelligence (AI) into healthcare offers an immense potential for enhancing disease diagnostics [1]. Within AI, speech and language technologies have emerged as valuable tools for investigating language production in patients with Parkinson's disease (PD). PD is associated with a variety of speech-related impairments, including dysarthria and various language disorders [2]. Early detection of subtle dysarthria in patterns within the prodromal stage of PD could enable earlier intervention with disease-modifying treatments [3].

Speech recordings play a critical role in identifying speech biomarkers and are usually evaluated using a diverse set of acoustic analysis methods [2]. Detection of PD based on audio signals typically relies on contrasting patient signals with those obtained from healthy controls (HC). Current state of the art methods often rely on high-performing self-supervised deep learning (DL). Recently, Nasersharif and Namvarpour categorized self-supervised models for robust speech representation learning into four groups [4]: recurrent models (typically employing modules like Long Short-Term Memory), Convolutional Neural Network (CNN)-based models, transformer-based models, and hybrid models that combine CNNs with transformers.

This study focuses on pre-trained model embeddings, particularly on the model Wav2Vec 1.0 (referred to as wav2vec1, wav2vec 1.0, or w2v1) [5], a CNN-based model that employs multi-layer CNNs to encode speech utterances, and the model Wav2Vec 2.0 (referred to as wav2vec2, wav2vec 2.0, or w2v2) [6], a representative set of hybrid models according to Nasersharif and Namvarpour’s design [4]. Previously, pre-trained embeddings have been shown to outperform traditional acoustic features in the detection of dysarthria [7]. Furthermore, Cai et al. [8], demonstrated the effectiveness of combining wav2vec 2.0 embeddings with machine learning models (e.g. Support Vector Machine (SVM), K-Nearest Neighbors (KNN), Decision Tree (DT), and Random Forest (RF) based methods) to classify normal and pathological speech, highlighting the benefits of audio augmentation. Wav2Vec 2.0 has also shown promise in detecting prodromal speech patterns in PD [3]. Alongside wav2vec 2.0, other digital architectures, such as HuBERT, TRILLsson, and Whisper, have also demonstrated superior performance in related classification tasks [3], [9], [10].

Pre-trained models and self-supervised learning approaches are commonly referred to as acoustic or speech foundation models (SFMs). Current research in this area is often hindered by inconsistent evaluation metrics and experimental protocols, making it difficult to draw meaningful comparisons across methods. A systematic evaluation of SFMs specifically for pathological speech detection remains largely unexplored. In 2025, BAHBench [11], A Unified Benchmark for Evaluating Bio-Acoustic Health with Acoustic Foundation Models, was introduced. While BAHBench represents a major step forward, its primary focus is limited to general bioacoustic datasets. While it includes a range of semantic and non-semantic tasks, only one dysarthric speech dataset was tested, which did not involve any individuals diagnosed with PD.

Wav2Vec 2.0 exists in multiple configurations. Javanmardi et al. [7] compared the base and large versions of the model, observing improved pathological speech detection performance when testing the large version. However, the base model, which features half as many transformer layers, and is trained on a smaller dataset, likely requires fewer computational resources. A comparison between the multilingual large-XLSR-53 variant (trained on 53 languages) and the monolingual English-trained base and large models, on tasks unrelated to PD, was presented in [12]. Across three benchmark tasks, the “XLSR-53” model consistently outperformed the others, while the “base” model showed the weakest performance. In a study focusing on PD, Favaro et al. [9] evaluated the Wav2Vec 2.0 base model across a wide range of languages from diverse language families. The model demonstrated consistently strong performance, indicating that effective detection of speech pathologies may be achievable without relying on multilingual models. This finding suggested that language-specific training may not be a strict requirement for robust pathological speech analysis, even in non-native or cross-linguistic contexts. On the other hand, the XLSR-53 model was pretrained on a much larger dataset, increasing the likelihood of exposure to pathological speech patterns during training, which may enhance its ability to detect such pathologies.

As highlighted in multiple studies [7], [11], [13], [14], [15], [16], [17], the choice of feature extraction layer in Wav2Vec 2.0 is critical for achieving optimal task-specific performance. To explore potential differences between the base and large variants, [11] tested both models and observed that both versions exhibited similar trends across tasks, with lower layers generally yielding better results and higher (semantic) layers showing a decline in performance. In a layer-wise analysis conducted on a Spanish dataset [14], XLSR and w2v2-base achieved comparable accuracy across layers, and both outperformed Whisper. Notably, XLSR demonstrated stable performance across middle layers, with the final layer consistently performing the worst. At least two recent publications have focused on evaluation of Wav2Vec 2.0 trained on English, its multilingual XLSR variant, and versions of XLSR-53 fine tuned on either English and Italian [16] or Spanish dysarthric speech [17]. These studies can serve as valuable reference points for understanding how different layers capture various types of acoustic information, and can provide practical insights into layer selection strategies and model variant choices. Importantly, the reported results are not solely determined by the model architecture or layer selection but are also influenced by the choice of classification method applied to the extracted representations, as shown by [15], which employed an SVM combined with SFM approach, and [14], employing a neural network (NN) consisting of a single hidden layer with 256 nodes and a binary output layer.

In addition to achieving a strong performance across various multilingual corpora and scenarios, wav2vec 2.0 has the ability to filter out pathology-unrelated fluctuations in spontaneous speech [18]. This capability could facilitate broader practical implementation, as spontaneous speech is easier and more convenient to record in real-world settings. In contrast, classical approaches can struggle to effectively capture pathology-specific cues in naturalistic speech contexts [18]. Another example of a recent comprehensive pipeline for analyzing speech and its linguistic properties in PD involved recording speech, transcribing it into text using Automatic Speech Recognition (ASR), and conducting further analyses such as part-of-speech tagging and syntactic complexity assessment [19]. ASR systems, including wav2vec-based ASR, are often utilized to assess speech intelligibility in PD [20], [21].

Wav2Vec 2.0 has established itself as one of the top-performing SFMs in dysarthria detection, however it cannot be regarded as universally superior across all tasks, with other SFMs showing competitive performance, including HuBERT, Whisper, WavLM, data2vec, and SEAMLESSM4T [10], [11], [14], [15]. Our focus on Wav2Vec 2.0 is motivated by three main factors: (1) it consistently delivers strong performance as a feature extractor, often outperforming other models; (2) it does not require any signal preprocessing, unlike several competing models that rely on spectrograms or filter bank representations; and (3) it supports a wide range of downstream tasks with minimal adaptation. Despite being smaller than some of the more recent architectures, Wav2Vec 2.0 remains a relatively complex model with substantial computational demands [12].

In our previous studies on the use of wav2vec 1.0, we demonstrated the potential for developing a universal speech-based PD evaluation model that was capable of outperforming traditional feature extraction methods in cross-database classification [22], [23]. Besides our own research, other studies investigating wav2vec for the detection of PD have focused exclusively on the transformer-based wav2vec 2.0. To our best knowledge, wav2vec is usually implicitly associated with wav2vec 2.0, however, it is critical to emphasize the existence of multiple competing versions, necessitating a thorough comparison to evaluate their respective advantages and limitations, particularly regarding their applicability across different speech modes in PD. This comparison aims to address and preclude potential misconceptions in the field.

We feel that the rapidly evolving scientific landscape would benefit from this more focused and detailed characterisation of the two versions of wav2vec with respect to their use in classifying normal versus PD-affected speech. This study provides a direct comparison of the two embedding architectures w2v1 and w2v2 using three different datasets, and employs a wide range of classification methods. Further, both wav2vec model embeddings are compared with traditional baseline features such as energy entropy or zero crossing rate. Beyond this, our aim was to explore the suitability of these models in various linguistic contexts, including spontaneous speech, non-spontaneous speech, and isolated vowels.

## 2 Methods

### 2.1 Speech corpuses

To demonstrate the proposed methods, address language differences, and evaluate the impact of various speech modes, we utilized audio recordings from three databases—an English database, a Spanish database, and a database focused on vowel recordings. In all proposed modeling tasks, the target variable was the prediction of PD based on audio recordings (PD vs. HC).

#### 2.1.1 English recordings

The dataset used for English recordings was the Mobile Device Voice Recordings at King's College London (MDVR-KCL) [24]. This dataset includes high-quality audio recordings with a sampling rate of 44.1 kHz, featuring both read text and spontaneous dialogue. For the read text, participants were asked to recite a fixed passage, "The North Wind and the Sun". Spontaneous dialogue was initiated by test administrators asking open-ended questions about topics such as local attractions, traffic, or personal interests. The dataset is slightly imbalanced, consisting of 21 HC and 16 participants with PD. In such cases, baseline accuracy, achieved by predicting all samples as HC, would yield 57 %. One PD participant provided only read text, resulting in 15 PD and 21 HC samples for the dialogue task. Clinical scores for the participants include the Hoehn and Yahr (H&Y) scale, with a mean score of 2.6 ± 0.72, and the Unified Parkinson's Disease Rating Scale (UPDRS) III, speech subscore (item 18), with a mean of 0.94 ± 0.93. Detailed demographic information for the participants is unavailable. This corpus features more complex speech tasks, but does not provide isolated vowels.

#### 2.1.2 Spanish recordings

The Spanish dataset used in this study was the PC-GITA, a well-known resource in PD research [25]. This dataset is balanced in terms of both sample size and gender distribution, consisting of 50 participants with PD (25 female, 25 male) and 50 HC (25 female, 25 male). For the PD group, the mean total UPDRS score was 37.7 ± 18.3, the mean UPDRS speech component was 1.3 ± 0.8, the mean H&Y scale was 2.2 ± 0.7, and the mean age was 61 ± 9.4 years. For the HC group, the mean age was 61 ± 9.5 years. The recordings had a sampling frequency of 44.1 kHz and included a variety of speech tasks, such as a read text, spontaneous monologues, and isolated vowels. For individuals with multiple recordings of the same task, only a single instance was selected for use in the proposed models, to prevent potential data leakage.

#### 2.1.3 Sustained vowel /a/ recordings

For sustained vowel analysis, we utilized a U.S.-based dataset [26]. This dataset consists of personal, telephone-collected recordings of the sustained vowel /a/ in natural settings (8 kHz sampling frequency), including samples from 50 participants with specialist-diagnosed PD and 50 HC. After pre-processing, the final study population consisted of 40 individuals with PD and 41 HC. The recordings represent prolonged enunciations of the vowel /a/ captured using participants' personal telephones. For the HC group, there were 16 males and 25 females with a mean age of 47.9 ± 14.5 years. For the PD group, there were 21 males and 19 females with a mean age of 66.6 ± 9.0 years and a mean H&Y score of 2.1 ± 0.4. Additionally, the vowel analysis was further extended using /a/ recordings from the PC-GITA dataset.

### 2.2 Traditional acoustic features

To establish a simple, straightforward, and easily interpretable baseline, we employed six traditional handcrafted acoustic features. These features were computed following the approach described in [27]. The feature extraction process involved two steps:

1. The audio signal was segmented into short-term, non-overlapping 50 ms frames. For each frame, six acoustic features were calculated (details can be found in Appendix B): Energy Entropy (A), Energy (B), Zero Crossing Rate (C), Spectral Rolloff (D), Spectral Centroid (E), and Spectral Flux (F). This resulted in six feature sequences representing the entire audio signal.
2. For each of the six feature sequences, a descriptive statistic was computed (Table 1), producing a single summary value for each feature. These six summary values collectively represent the final feature set that characterizes the input speech signal.

**Table 1:** List of calculated traditional features.

Feature	Statistic	Domain
EnergyEntropy	std	time
SignalEnergy	std/mean	time
ZeroCrossingRate	std	time
SpectralRolloff	std	spectral
SpectralCentroid	std	spectral
SpectralFlux	std/mean	spectral

### 2.3 Speech pre-trained embeddings

**Fig. A1:**
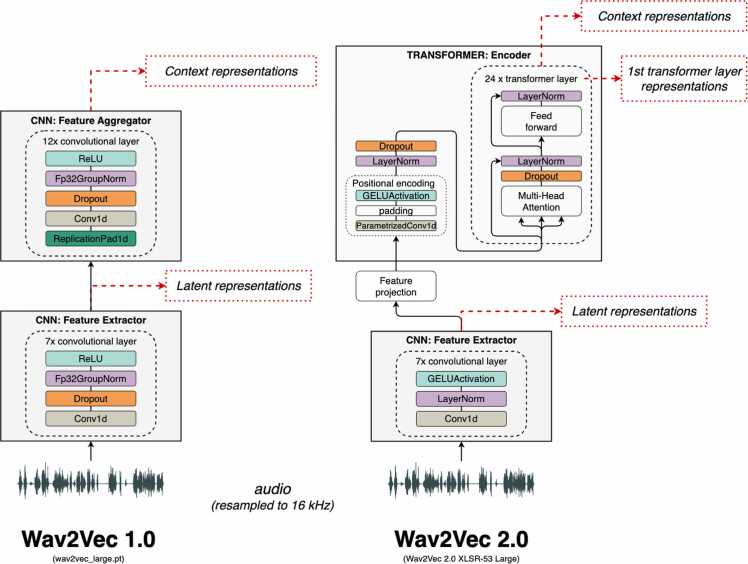
Two architecture versions of the Wav2Vec used

We focused on the two previously described architectures of the Wav2Vec model, 1.0 and 2.0 (Appendix A Fig. A1). Wav2Vec-based features are inherently non-interpretable but serve as universal representations, eliminating the need for handcrafted feature engineering. As a preprocessing step, all speech signals were resampled to 16 kHz using *torchaudio.transforms.Resample* [28]. Both Wav2Vec architectures were originally trained in a self-supervised manner, optimizing a contrastive loss function. The extracted features were acoustic-level. We did not perform fine-tuning on the Wav2Vec architectures.

#### 2.3.1 Wav2Vec 1.0

We utilized the original Wav2Vec model [5]. This is a CNN-based architecture that employs multi-layer convolutional NNs to encode speech utterances. Wav2Vec 1.0 comprises two cascaded CNN modules, a feature extractor and a feature aggregator. The feature extractor refines raw audio signals into preliminary representations, while the feature aggregator integrates these representations into higher-level latent variables that capture contextual semantic relationships among audio features [29].

For our analysis, we employed the "wav2vec_large" variant, which has an expanded capacity due to its larger context network, consisting of twelve layers [30]. This model was pre-trained on the LibriSpeech dataset, comprising 960 h of English speech sampled at 16 kHz. We extracted representations from two points in the model: (1) the output of the feature extractor (FE) and (2) the combined output of the feature extractor and aggregator (FEA).

#### 2.3.2 Wav2Vec 2.0

Wav2Vec 2.0 is a hybrid model that integrates CNNs with transformers. Each transformer layer processes the input through self-attention mechanisms and feed-forward networks, producing intermediate representations that encapsulate progressively more abstract and semantically rich information. As a result, the outputs from different transformer layers can encode diverse levels of information about the input audio, ranging from phonetic details to higher-level semantic patterns.

For our analysis, we employed the Wav2Vec 2.0 XLSR-53 model, a multilingual variant pre-trained on 53 languages, including English and Spanish [31], [32]. We focused on extracting representations from several key points in the CNN and transformer stack: the feature extractor, the first transformer layer and the last hidden layer. By analyzing these layers, we aimed to understand the evolution of speech representations across the network.

#### 2.3.3 Embeddings aggregation

The obtained Wav2Vec representations were converted into fixed-sized, utterance-level feature vectors using mean aggregation. Vetráb and Gosztolya explored various aggregation techniques for Wav2Vec 2.0 deep embeddings, evaluating 11 different functions, and confirming that mean aggregation was an effective choice [33]. For Wav2Vec 1.0, the aggregation resulted in 512-dimensional vectors, while the Wav2Vec 2.0 dimensions varied depending on the selected layer, yielding either 1024- (transformer) or 512-dimensional (feature extractor) vectors. To further process the high-dimensional representations, we applied Principal Component Analysis (PCA) to decorrelate the feature space. From each Wav2Vec representation, we extracted the first 30 PCA components for subsequent analysis.

#### 2.3.4 Embeddings fusion

To investigate whether each representation captures complementary information from the input signal, we combined Wav2Vec 1.0 and Wav2Vec 2.0 embeddings at selected layers. We adopted an early fusion strategy, as described in [34], whereby feature vectors are concatenated along the feature dimension to form a single composite representation.

### 2.4 Classification methods

The feature representations generated for each audio recording (w2v1, w2v2 and baselines) were used as input features for a variety of classification methods implemented in the Python library scikit-learn [35]. Specifically, we evaluated the following classifiers: Naive Bayes (NB; Gaussian NB), Support Vector Machine (SVM; with a linear kernel and probabilistic output), Decision Tree (DT; using default parameters), K-Nearest Neighbors (KNN; with n_neighbors=5), Logistic Regression (LR; with max_iter=1000), and Random Forest (RF; with n_estimators=100). Each classifier was initially trained using default or standard hyperparameter settings.

### 2.5 Metrics and evaluation strategy

To assess the performance of the HC vs. PD classification models, we used the following metrics (True Positives, TP; True Negatives, TN; False Positives, FP; False Negatives, FN):

• *Accuracy:* Defined as $\frac{\mathrm{TP}+\mathrm{TN}}{\mathrm{TP}+\mathrm{FN}+\mathrm{TN}+\mathrm{FP}}$, the accuracy metric measures the proportion of correctly classified instances in the dataset.
• *Sensitivity (Recall):* Calculated as $\frac{\mathrm{TP}}{\mathrm{TP}+\mathrm{FN}}$, sensitivity evaluates the model's ability to identify positive instances correctly.
• *Specificity:* Given by $\frac{\mathrm{TN}}{\mathrm{TN}+\mathrm{FP}}$, specificity quantifies the model's ability to correctly identify negative instances.
• *Area Under the Curve (AUC):* The area under the receiver operating characteristic (ROC) curve was computed using the roc_auc_score function from scikit-learn. This metric provides a single scalar value to summarize the trade-off between sensitivity and specificity across different classification thresholds.
• *Precision:* Defined as $\frac{\mathrm{TP}}{\mathrm{TP}+\mathrm{FP}}$, the precision metric measures the proportion of correctly predicted positive instances out of all predicted positives. This metric is particularly important in imbalanced datasets to ensure that the model does not generate a high number of false positives.
• *F1 Score*: The F1 score is the harmonic mean of precision and recall, calculated as $F1\;=\;2\;\times \;\frac{\mathrm{precision}\times \mathrm{recall}}{\mathrm{precision}+\mathrm{recall}}$.
• *Matthews Correlation Coefficient (MCC)*: MCC is a balanced metric defined as $\mathrm{MCC}=\;\frac{\left(\mathrm{TP}\times \mathrm{TN})\;-\;(\mathrm{FP}\times \mathrm{FN}\right)}{\sqrt{\left(\mathrm{TP}+\mathrm{FP})(\mathrm{TP}+\mathrm{FN})(\mathrm{TN}+\mathrm{FP})(\mathrm{TN}+\mathrm{FN}\right)}}$, producing a score between −1 and + 1, where + 1 indicates perfect prediction, 0 represents no better than random guessing, and −1 signifies complete disagreement between predictions and actual values.

We used a stratified 10-fold cross-validation strategy to ensure robust performance evaluation. All classifier evaluations were conducted within individual datasets (intra-dataset evaluations), without performing cross-database evaluations.

### 2.6 Ranking speech representations using a multicriteria approach

To identify the optimal speech representation for generating the best classification performance across different speech modes, we employed a Multi-Criteria Decision Analysis (MCDA). This method focuses on ranking alternatives based on multiple criteria simultaneously. By evaluating alternatives across several metrics derived from machine learning models, MCDA provided a structured framework for comparing and prioritizing options. We adopted the Technique for Order of Preference by Similarity to Ideal Solution (TOPSIS), a multicriteria approach, for ranking machine learning classifiers as outlined in [36].

#### 2.6.1 TOPSIS MCDA

TOPSIS was selected to be the MCDA method in this study due to its suitability for machine learning tasks and its ability to incorporate the Euclidean distance from an ideal solution. TOPSIS is widely used in biomedical engineering for healthcare technology assessment, such as selecting the most appropriate medical devices from a given set of performance metrics [37]. For each speech mode, six speech representations were evaluated using the TOPSIS method, with each representation characterized by seven metrics, including accuracy, sensitivity, specificity, AUC, precision, F1 score, and MCC, representing the average performance across multiple classifiers. TOPSIS identified the optimal alternative by minimizing the Euclidean distance to the positive ideal solution (PIS) while maximizing the distance from the negative ideal solution (NIS).

To ensure a fair comparison in our TOPSIS-based ranking, all classifiers were evaluated using a common set of default hyperparameters. This approach prevented any single model–representation pair from gaining an artificial advantage through tuning, allowing direct comparison of their inherent representational quality.

To implement TOPSIS, we first normalized the performance values of each alternative to a scale between 0 and 1 and assigned equal weights to all metrics. These weighted values were then used to compute the PIS, representing the best possible performance, and the NIS, representing the worst possible performance. The Euclidean distances from each alternative to the PIS and NIS were calculated, and alternatives were ranked based on their proximity to the PIS. Additional details regarding the implementation can be found in Appendix C. In this study, the runtime of each speech representation was not included as a criterion for TOPSIS, as it heavily depends on hardware configurations.

### 2.7 Hyperparameter optimization

To illustrate the potential gains achievable through model-specific calibration, we additionally performed an extensive hyperparameter optimization outside of the TOPSIS framework. We systematically tuned hyperparameters for each classifier using a 5-fold Grid Search CV over selected parameter grids. For the NB model, we varied the additive smoothing parameter var_smoothing across a log-spaced range from 1e-11–1e-7 to ensure numerical stability. For the SVM classifier, we tuned the regularization parameter C with values [0.01, 0.1, 1, 10, 100] and evaluated both linear and radial basis function kernels. In the DT model, we explored maximum tree depths [None, 5, 10, 20], minimum samples required to split a node [2, 5, 10, 20], and minimum samples required at a leaf [1, 2, 4, 8] to control tree complexity. The KNN classifier was optimized by testing different numbers of neighbors [3, 5, 7, 9, 11, 15] and comparing uniform and distance-based weighting schemes. For LR, we tuned the inverse regularization strength C over [0.001, 0.01, 0.1, 1, 10] and used scikit-learn solvers including lbfgs, liblinear, and saga. Finally, for the RF model, we adjusted the number of trees [50, 100, 200], maximum depth [10, 20, None], minimum samples to split a node [2, 5, 10], minimum samples per leaf [1, 2, 4], and tested different strategies for selecting the number of features at each split including sqrt, log2, and all features. For each model, the best hyperparameter combination was selected based on its cross-validated accuracy.

## 3 Results

### 3.1 Ranking the speech representations using the multicriteria approach

The TOPSIS method was applied to compare the performance of Wav2Vec 1.0, Wav2Vec 2.0, and baseline features for classification of PD patients and HC, ranking the representations across all considered speech modes. According to the TOPSIS results, Wav2Vec 2.0 was identified as the best-performing approach overall. Specifically, the first transformer layer of Wav2Vec 2.0 demonstrated the highest performance on connected speech tasks, i.e. reading text and monologue, across datasets, while its feature extractor proved most effective for vowel-based tasks.

Wav2Vec 1.0 ranked second among the representations, with its feature extractor performing better for vowel-based tasks and a combination of its feature extractor and aggregator showing improved results for connected speech modes. An exception to these general trends was observed in the dialogue speech mode within the English dataset, which showed significantly different behavior across the representations, where baselines features performed the best. The TOPSIS scoring results are summarized in Table 2, while a comprehensive TOPSIS table, including detailed metrics, is provided in Appendix D.1.

**Table 2:** MCDA TOPSIS ranking. Topsis incorporated all metrics across classifiers, including accuracy, sensitivity, specificity, AUC, precision, F1 score, and MCC. Green highlights indicate detected trends across datasets and modes involving connected speech tasks, while blue highlights mark trends specific to the simpler vowel-based tasks.

w2v2–1T: first transformer layer; w2v2-LH: last hidden layer; w2v2-FE: feature extraction; w2v1-FE: feature extraction; w2v1-FEA: feature extraction and aggregation

### 3.2 Runtime and PCA

**Fig. 1:**
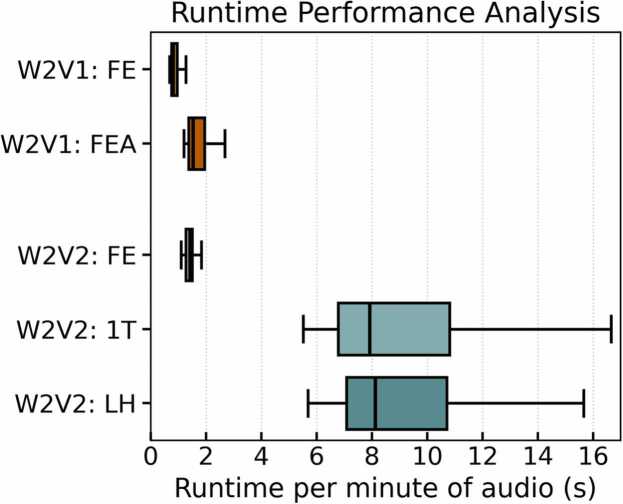
Boxplot of runtime per audio recording, normalized to one minute of audio for fair comparison across different sample lengths. The reported runtime refers specifically to the feature extraction step, not to model training or classification. Outliers were removed, including a Wav2Vec 2.0 instance exceeding 200 s. All measurements were performed on a MacBook air with an M1 chip and 8 GB of RAM.

Wav2Vec 1.0 was the fastest implementation in terms of computation time, highlighting its potential for time-sensitive applications (Fig. 1).

**Fig. 2:**
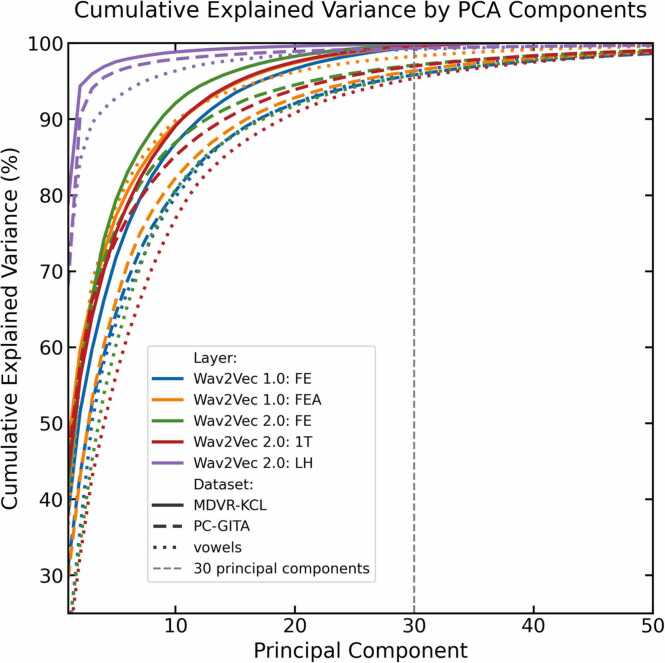
Plot of the cumulative explained variance as a function of the number of PCA components. The first 30 components captured approximately 95 % of the original variance across all datasets.

For classification purposes, we computed the first 30 PCA components for all Wav2Vec representations. Fig. 2 illustrates the cumulative explained variance as a function of the number of PCA components.

### 3.3 Developed classification models across two speech modes using the English dataset

#### 3.3.1 Classifiers used in TOPSIS

The performance of different classifiers was evaluated across two speech modes, read text and spontaneous dialogue, using the English dataset. For the read text mode, the best results across multiple metrics were achieved using the w2v2–1T and w2v1-FEA. However, there were performance variations observed across individual layers of the w2v2, with the w2v2-LH layer showing the weakest performance. Sensitivity remained low across all representations. Detailed classifier performance metrics are presented in Table 3. Notably, for spontaneous dialogue (Table 4), traditional baseline features demonstrated the best overall performance, outperforming both Wav2Vec representations.

**Table 3:** Classifier performance metrics for read text from the English dataset, which included 21 HC and 16 PD samples in an imbalanced distribution.

	**classifier**	**accuracy**	**sens.**	**spec.**	**precision**	**F1**	**MCC**	**AUC**
**wav2vec 1.0 feature extractor**	DT	0.62	0.60	0.65	0.60	0.58	0.25	0.63
	KNN	0.73	0.35	1.00	0.50	0.40	0.37	0.90
	LR	0.58	0.00	1.00	0.00	0.00	0.00	0.85
	NB	0.73	0.60	0.82	0.70	0.60	0.45	0.74
	RF	0.80	0.60	0.95	0.75	0.63	0.58	0.88
	SVM	0.58	0.00	1.00	0.00	0.00	0.00	0.33
	**mean ± std**	**0.67 ± 0.10**	**0.36 ± 0.29**	**0.90 ± 0.14**	**0.43 ± 0.34**	**0.37 ± 0.30**	**0.28 ± 0.24**	**0.72 ± 0.22**
**wav2vec 1.0 feature extractor + aggregator**	DT	0.74	0.65	0.80	0.72	0.66	0.47	0.73
	KNN	0.71	0.30	1.00	0.50	0.37	0.33	0.73
	LR	0.74	0.45	0.95	0.60	0.50	0.42	0.73
	NB	0.76	0.55	0.90	0.70	0.58	0.48	0.79
	RF	0.79	0.60	0.95	0.75	0.65	0.57	0.80
	SVM	0.73	0.35	1.00	0.50	0.40	0.37	0.75
	**mean ± std**	**0.75 ± 0.03**	**0.48 ± 0.14**	**0.93 ± 0.08**	**0.63 ± 0.11**	**0.53 ± 0.13**	**0.44 ± 0.09**	**0.75 ± 0.03**
**wav2vec 2.0 feature extractor**	DT	0.58	0.45	0.65	0.42	0.41	0.09	0.55
	KNN	0.66	0.20	1.00	0.30	0.23	0.22	0.67
	LR	0.58	0.00	1.00	0.00	0.00	0.00	0.75
	NB	0.70	0.75	0.67	0.68	0.66	0.45	0.80
	RF	0.70	0.55	0.80	0.52	0.51	0.37	0.85
	SVM	0.58	0.00	1.00	0.00	0.00	0.00	0.38
	**mean ± std**	**0.63 ± 0.06**	**0.33 ± 0.31**	**0.85 ± 0.17**	**0.32 ± 0.28**	**0.30 ± 0.27**	**0.19 ± 0.19**	**0.67 ± 0.18**
**wav2vec 2.0 last hidden layer**	DT	0.62	0.45	0.72	0.57	0.48	0.20	0.58
	KNN	0.66	0.55	0.78	0.60	0.53	0.36	0.66
	LR	0.58	0.00	1.00	0.00	0.00	0.00	0.38
	NB	0.52	0.35	0.65	0.40	0.35	0.02	0.59
	RF	0.63	0.35	0.83	0.55	0.42	0.22	0.64
	SVM	0.58	0.00	1.00	0.00	0.00	0.00	0.43
	**mean ± std**	**0.59 ± 0.05**	**0.28 ± 0.23**	**0.83 ± 0.15**	**0.35 ± 0.28**	**0.30 ± 0.24**	**0.13 ± 0.15**	**0.55 ± 0.12**
**wav2vec 2.0 first transformer layer**	DT	0.69	0.50	0.85	0.62	0.51	0.39	0.68
	KNN	0.61	0.15	0.95	0.20	0.17	0.10	0.76
	LR	0.70	0.65	0.75	0.60	0.59	0.42	0.95
	NB	0.70	0.75	0.67	0.68	0.66	0.45	0.81
	RF	0.82	0.75	0.87	0.78	0.73	0.65	0.95
	SVM	0.73	0.65	0.80	0.62	0.60	0.47	0.90
	**mean ± std**	**0.71 ± 0.07**	**0.58 ± 0.23**	**0.81 ± 0.10**	**0.58 ± 0.20**	**0.54 ± 0.20**	**0.41 ± 0.18**	**0.84 ± 0.11**
**baselines**	DT	0.44	0.30	0.52	0.28	0.27	−0.19	0.41
	KNN	0.58	0.40	0.72	0.38	0.37	0.12	0.65
	LR	0.73	0.60	0.82	0.60	0.58	0.42	0.79
	NB	0.70	0.50	0.87	0.60	0.50	0.40	0.73
	RF	0.66	0.55	0.77	0.57	0.51	0.34	0.56
	SVM	0.73	0.60	0.82	0.60	0.58	0.42	0.76
	**mean ± std**	**0.64 ± 0.11**	**0.49 ± 0.12**	**0.75 ± 0.13**	**0.51 ± 0.14**	**0.47 ± 0.13**	**0.25 ± 0.25**	**0.65 ± 0.15**

**Table 4:** Classifier performance metrics for dialogue from the English dataset, which included 21 HC and 15 PD samples in an imbalanced distribution.

	**classifier**	**accuracy**	**sens.**	**spec.**	**precision**	**F1**	**MCC**	**AUC**
**wav2vec 1.0 feature extractor**	DT	0.72	0.75	0.70	0.65	0.66	0.47	0.73
	KNN	0.72	0.45	0.92	0.50	0.47	0.37	0.61
	LR	0.59	0.00	1.00	0.00	0.00	0.00	0.82
	NB	0.71	0.60	0.83	0.53	0.53	0.44	0.75
	RF	0.77	0.65	0.88	0.58	0.60	0.53	0.75
	SVM	0.59	0.00	1.00	0.00	0.00	0.00	0.62
	**mean ± std**	**0.68 ± 0.07**	**0.41 ± 0.33**	**0.89 ± 0.11**	**0.38 ± 0.30**	**0.38 ± 0.30**	**0.30 ± 0.24**	**0.71 ± 0.08**
**wav2vec 1.0 feature extractor + aggregator**	DT	0.48	0.35	0.58	0.23	0.27	−0.07	0.47
	KNN	0.68	0.55	0.82	0.50	0.50	0.35	0.64
	LR	0.72	0.50	0.92	0.50	0.48	0.42	0.79
	NB	0.70	0.65	0.78	0.58	0.57	0.45	0.70
	RF	0.68	0.55	0.83	0.48	0.48	0.38	0.85
	SVM	0.72	0.50	0.92	0.50	0.48	0.42	0.69
	**mean ± std**	**0.66 ± 0.09**	**0.52 ± 0.10**	**0.81 ± 0.12**	**0.47 ± 0.12**	**0.46 ± 0.10**	**0.32 ± 0.20**	**0.69 ± 0.13**
**wav2vec 2.0 feature extractor**	DT	0.52	0.55	0.57	0.40	0.40	0.13	0.56
	KNN	0.66	0.40	0.87	0.45	0.40	0.27	0.68
	LR	0.59	0.00	1.00	0.00	0.00	0.00	0.72
	NB	0.63	0.70	0.62	0.53	0.57	0.32	0.75
	RF	0.62	0.50	0.77	0.38	0.40	0.27	0.77
	SVM	0.59	0.00	1.00	0.00	0.00	0.00	0.42
	**mean ± std**	**0.60 ± 0.05**	**0.36 ± 0.29**	**0.80 ± 0.19**	**0.29 ± 0.23**	**0.29 ± 0.24**	**0.16 ± 0.14**	**0.65 ± 0.14**
**wav2vec 2.0 last hidden layer**	DT	0.68	0.60	0.72	0.53	0.54	0.32	0.66
	KNN	0.44	0.10	0.67	0.13	0.11	−0.24	0.38
	LR	0.59	0.00	1.00	0.00	0.00	0.00	0.33
	NB	0.39	0.15	0.55	0.20	0.17	−0.31	0.40
	RF	0.48	0.20	0.67	0.25	0.20	−0.13	0.44
	SVM	0.59	0.00	1.00	0.00	0.00	0.00	0.53
	**mean ± std**	**0.53 ± 0.11**	**0.18 ± 0.22**	**0.77 ± 0.19**	**0.19 ± 0.20**	**0.17 ± 0.20**	**−0.06 ± 0.22**	**0.45 ± 0.12**
**wav2vec 2.0 first transformer layer**	DT	0.57	0.45	0.67	0.32	0.36	0.10	0.56
	KNN	0.68	0.55	0.82	0.53	0.51	0.37	0.66
	LR	0.70	0.65	0.77	0.53	0.57	0.40	0.73
	NB	0.67	0.70	0.67	0.53	0.57	0.37	0.83
	RF	0.68	0.55	0.82	0.45	0.47	0.36	0.80
	SVM	0.63	0.65	0.67	0.50	0.55	0.30	0.70
	**mean ± std**	**0.65 ± 0.05**	**0.59 ± 0.09**	**0.73 ± 0.08**	**0.48 ± 0.09**	**0.50 ± 0.08**	**0.31 ± 0.11**	**0.71 ± 0.10**
**baselines**	DT	0.62	0.50	0.73	0.45	0.45	0.25	0.62
	KNN	0.58	0.45	0.68	0.42	0.40	0.17	0.68
	LR	0.78	0.70	0.88	0.78	0.68	0.61	0.83
	NB	0.72	0.60	0.82	0.73	0.62	0.45	0.81
	RF	0.66	0.50	0.78	0.53	0.48	0.31	0.63
	SVM	0.76	0.65	0.88	0.68	0.62	0.56	0.83
	**mean ± std**	**0.69 ± 0.08**	**0.57 ± 0.10**	**0.80 ± 0.08**	**0.60 ± 0.15**	**0.54 ± 0.11**	**0.39 ± 0.18**	**0.73 ± 0.10**

When comparing Wav2Vec-based approaches across non-spontaneous and spontaneous speech modes, clear differences in performance were observed. For non-spontaneous speech, the w2v1-FEA achieved an accuracy of 0.75 and an AUC of 0.75. In contrast, for spontaneous dialogue, its performance decreased to an accuracy of 0.66 and an AUC of 0.69. Similarly, the w2v2–1T yielded an accuracy of 0.71 and an AUC of 0.84 for non-spontaneous speech, while its performance dropped to an accuracy of 0.65 and an AUC of 0.71 for spontaneous dialogue. These results highlight a consistent trend of higher performance in non-spontaneous speech compared to spontaneous dialogue, with differences in accuracy ranging from 6 % to 9 % and differences in the AUC ranging from 4 % to 13 %, depending on the representation.

Performance variability was observed across both representations and classifiers throughout the English dataset. Importantly, LR and SVM classifiers failed in several instances, misclassifying all test examples as HC, resulting in a sensitivity of 0 and specificity of 1. Appendix D2 and Appendix D3 provide a comparison of PCA-reduced Wav2Vec embeddings for both speech modes.

#### 3.3.2 Optimized classifiers

Fine-tuning classifiers led to consistent improvements in accuracy. For brevity, we report results only for the top two representations identified by the TOPSIS. For w2v1-FEA in the read text task, fine-tuning increased the mean accuracy from 0.75 to 0.78, with a maximum accuracy gain from 0.79 to 0.86. For w2v2–1T, fine-tuning improved the mean accuracy from 0.71 to 0.74, with the best accuracy rising from 0.82 to 0.86. For the dialogue task, fine-tuning raised the mean baseline accuracy from 0.69 to 0.70, with a maximum improvement from 0.70 to 0.76. For w2v2–1T, mean accuracy increased from 0.65 to 0.70, and the best accuracy improved from 0.70 to 0.80. Even after tuning, w2v2-LH showed low performance, falling below the baseline threshold, with a mean accuracy of 0.52 and an AUC of 0.42.

**Fig. 3:**
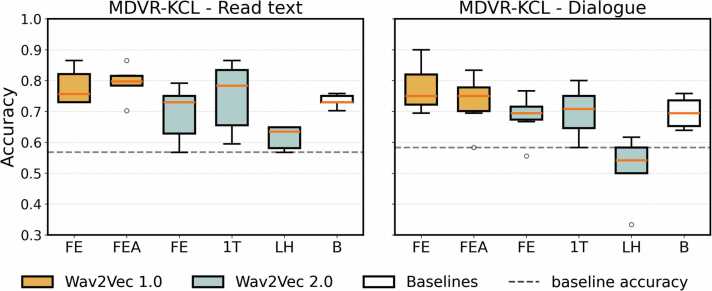
Distributions of accuracies across fine-tuned classifiers for the English dataset.

Wav2Vec 1.0 showed significant gains after fine-tuning for both tasks, with the highest observed accuracy being 0.90 for the DT approach in the dialogue task. Distributions of accuracies across fine-tuned classifiers for each representation can be found (Fig. 3). The best classification results obtained for each representation can be found in Table 5.

**Table 5:** Classification metrics of the best fine-tuned classifier for each feature extraction method.

**mode**	**representation**	**classifier**	**accuracy**	**sens.**	**spec.**	**AUC**	**F1**
**read text**	w2v1-FE	RF	0.86	0.75	0.95	0.84	0.83
	w2v1-FEA	SVM	0.86	0.75	0.95	0.87	0.83
	w2v2-FE	SVM	0.79	0.60	0.90	0.85	0.61
	w2v2-1T	SVM	0.86	0.81	0.90	0.89	0.84
	w2v2-LH	NB	0.65	0.50	0.76	0.63	0.55
	baselines	LR	0.76	0.60	0.88	0.83	0.60
**dialogue**	w2v1-FE	DT	0.90	0.80	1.00	0.91	0.87
	w2v1-FEA	SVM	0.83	0.67	0.95	0.83	0.77
	w2v2-FE	NB	0.77	0.90	0.65	0.70	0.80
	w2v2-1T	NB	0.80	0.85	0.75	0.78	0.83
	w2v2-LH	KNN	0.62	0.20	0.90	0.62	0.22
	baselines	LR	0.76	0.60	0.87	0.86	0.62

w2v2–1T: first transformer layer; w2v2-LH: last hidden layer; w2v2-FE: feature extraction; w2v1-FE: feature extraction; w2v1-FEA: feature extraction and aggregation

#### 3.3.3 Embeddings fusion

As shown in Table 6, combined embeddings (w2v1+w2v2) selected from the best performing layers of Wav2Vec 1.0 and 2.0 did not result in any significant performance improvements when compared with the best fine-tuned classifiers. In the case of the dialogue task, accuracy was observed to decrease.

**Table 6:** MDVR-KCL: classification performance (fine-tuned) using early fusion of Wav2Vec embeddings.

**mode**	**representation**	**classifier**	**accuracy**	**sens.**	**spec.**	**AUC**	**F1**
**read text**	w2v1-FEA	SVM	0.86	0.75	0.95	0.87	0.83
	w2v2-1T	SVM	0.86	0.81	0.90	0.89	0.84
	v1 +v2	RF	0.87	0.70	1.00	0.90	0.73
**dialogue**	w2v1-FEA	SVM	0.83	0.67	0.95	0.83	0.77
	w2v2-1T	NB	0.80	0.85	0.75	0.78	0.83
	v1 +v2	LR	0.75	0.73	0.76	0.83	0.71

w2v1-FEA: feature extraction and aggregation; w2v2–1T: first transformer layer

### 3.4 Developed classification models across two speech modes using the PC-GITA dataset

#### 3.4.1 Classifiers used in TOPSIS

The performance of different classifiers was evaluated across read text (Table 7) and spontaneous monologue (Table 8) using the PC-GITA dataset. The w2v2–1T delivered the best overall performance for both speech modes, consistently outperforming other representations. Within w2v1, the FEA significantly enhanced performance compared to the FE.

**Table 7:** Classifier performance metrics for read text from the PC-GITA dataset, which included 50 HC and 50 PD samples in a balanced distribution.

	**classifier**	**accuracy**	**sens.**	**spec.**	**precision**	**F1**	**MCC**	**AUC**
**wav2vec 1.0 feature extractor**	DT	0.75	0.84	0.66	0.74	0.77	0.54	0.75
	KNN	0.75	0.60	0.90	0.88	0.69	0.54	0.83
	LR	0.69	0.58	0.80	0.76	0.64	0.40	0.75
	NB	0.74	0.62	0.86	0.84	0.70	0.51	0.79
	RF	0.85	0.82	0.88	0.89	0.84	0.72	0.90
	SVM	0.66	0.50	0.82	0.75	0.59	0.35	0.34
	**mean ± std**	**0.74 ± 0.07**	**0.66 ± 0.14**	**0.82 ± 0.09**	**0.81 ± 0.07**	**0.70 ± 0.09**	**0.51 ± 0.13**	**0.73 ± 0.20**
**wav2vec 1.0 feature extractor + aggregator**	DT	0.73	0.78	0.68	0.72	0.74	0.47	0.73
	KNN	0.75	0.60	0.90	0.88	0.69	0.54	0.81
	LR	0.75	0.68	0.82	0.80	0.71	0.52	0.87
	NB	0.71	0.64	0.78	0.75	0.67	0.44	0.79
	RF	0.78	0.78	0.78	0.79	0.78	0.57	0.87
	SVM	0.81	0.76	0.86	0.86	0.78	0.65	0.90
	**mean ± std**	**0.76 ± 0.04**	**0.71 ± 0.08**	**0.80 ± 0.08**	**0.80 ± 0.06**	**0.73 ± 0.04**	**0.53 ± 0.07**	**0.83 ± 0.06**
**wav2vec 2.0 feature extractor**	DT	0.57	0.56	0.58	0.59	0.56	0.15	0.57
	KNN	0.75	0.66	0.84	0.83	0.71	0.53	0.83
	LR	0.68	0.76	0.60	0.70	0.71	0.37	0.77
	NB	0.68	0.76	0.60	0.69	0.71	0.37	0.73
	RF	0.82	0.80	0.84	0.88	0.81	0.67	0.89
	SVM	0.69	0.78	0.60	0.71	0.73	0.39	0.76
	**mean ± std**	**0.70 ± 0.08**	**0.72 ± 0.09**	**0.68 ± 0.13**	**0.73 ± 0.10**	**0.70 ± 0.08**	**0.42 ± 0.17**	**0.76 ± 0.11**
**wav2vec 2.0 last hidden layer**	DT	0.58	0.56	0.60	0.54	0.54	0.15	0.58
	KNN	0.56	0.48	0.64	0.58	0.51	0.13	0.62
	LR	0.40	0.32	0.48	0.39	0.34	−0.21	0.38
	NB	0.58	0.44	0.72	0.60	0.49	0.17	0.60
	RF	0.73	0.76	0.70	0.76	0.74	0.48	0.80
	SVM	0.45	0.18	0.72	0.33	0.21	−0.12	0.56
	**mean ± std**	**0.55 ± 0.12**	**0.46 ± 0.20**	**0.64 ± 0.09**	**0.53 ± 0.15**	**0.47 ± 0.18**	**0.10 ± 0.25**	**0.59 ± 0.13**
**wav2vec 2.0 first transformer layer**	DT	0.78	0.80	0.76	0.78	0.78	0.58	0.78
	KNN	0.76	0.68	0.84	0.84	0.73	0.55	0.78
	LR	0.82	0.80	0.84	0.86	0.82	0.66	0.89
	NB	0.68	0.80	0.56	0.67	0.72	0.37	0.74
	RF	0.79	0.78	0.80	0.85	0.78	0.61	0.86
	SVM	0.82	0.80	0.84	0.86	0.81	0.66	0.88
	**mean ± std**	**0.78 ± 0.05**	**0.78 ± 0.05**	**0.77 ± 0.11**	**0.81 ± 0.07**	**0.77 ± 0.04**	**0.57 ± 0.11**	**0.82 ± 0.06**
**baselines**	DT	0.55	0.62	0.48	0.54	0.57	0.10	0.55
	KNN	0.57	0.60	0.54	0.55	0.57	0.14	0.61
	LR	0.60	0.62	0.58	0.61	0.59	0.20	0.63
	NB	0.51	0.66	0.36	0.49	0.56	0.05	0.50
	RF	0.61	0.62	0.60	0.58	0.58	0.23	0.58
	SVM	0.60	0.60	0.60	0.62	0.59	0.21	0.65
	**mean ± std**	**0.57 ± 0.04**	**0.62 ± 0.02**	**0.53 ± 0.09**	**0.57 ± 0.05**	**0.58 ± 0.01**	**0.15 ± 0.07**	**0.59 ± 0.05**

**Table 8:** Classifier performance metrics for monologue from the PC-GITA dataset, which included 50 HC and 50 PD samples in a balanced distribution.

	**classifier**	**accuracy**	**sens.**	**spec.**	**precision**	**F1**	**MCC**	**AUC**
**wav2vec 1.0 feature extractor**	DT	0.66	0.64	0.68	0.69	0.64	0.34	0.66
	KNN	0.75	0.68	0.82	0.79	0.72	0.51	0.83
	LR	0.69	0.62	0.76	0.73	0.65	0.39	0.80
	NB	0.75	0.68	0.82	0.79	0.72	0.51	0.83
	RF	0.85	0.84	0.86	0.86	0.85	0.71	0.92
	SVM	0.65	0.52	0.78	0.73	0.58	0.33	0.39
	**mean ± std**	**0.73 ± 0.07**	**0.66 ± 0.10**	**0.79 ± 0.06**	**0.77 ± 0.06**	**0.69 ± 0.09**	**0.47 ± 0.14**	**0.74 ± 0.19**
**wav2vec 1.0 feature extractor + aggregator**	DT	0.68	0.62	0.74	0.73	0.65	0.38	0.68
	KNN	0.77	0.72	0.82	0.83	0.75	0.56	0.82
	LR	0.77	0.74	0.80	0.81	0.76	0.56	0.86
	NB	0.73	0.68	0.78	0.73	0.69	0.47	0.82
	RF	0.81	0.78	0.84	0.83	0.80	0.63	0.87
	SVM	0.80	0.74	0.86	0.85	0.77	0.62	0.90
	**mean ± std**	**0.76 ± 0.05**	**0.71 ± 0.06**	**0.81 ± 0.04**	**0.80 ± 0.05**	**0.74 ± 0.06**	**0.54 ± 0.10**	**0.82 ± 0.08**
**wav2vec 2.0 feature extractor**	DT	0.76	0.74	0.78	0.79	0.75	0.54	0.76
	KNN	0.78	0.74	0.82	0.83	0.76	0.58	0.83
	LR	0.69	0.74	0.64	0.68	0.70	0.40	0.79
	NB	0.69	0.78	0.60	0.67	0.71	0.40	0.75
	RF	0.84	0.82	0.86	0.89	0.83	0.71	0.88
	SVM	0.69	0.80	0.58	0.67	0.72	0.41	0.79
	**mean ± std**	**0.74 ± 0.06**	**0.77 ± 0.04**	**0.71 ± 0.12**	**0.75 ± 0.09**	**0.75 ± 0.05**	**0.51 ± 0.13**	**0.80 ± 0.05**
**wav2vec 2.0 last hidden layer**	DT	0.68	0.66	0.70	0.71	0.67	0.37	0.68
	KNN	0.61	0.54	0.68	0.65	0.56	0.24	0.66
	LR	0.57	0.52	0.62	0.60	0.53	0.16	0.63
	NB	0.60	0.40	0.80	0.66	0.47	0.22	0.68
	RF	0.70	0.70	0.70	0.72	0.69	0.42	0.81
	SVM	0.53	0.08	0.98	0.35	0.13	0.10	0.39
	**mean ± std**	**0.62 ± 0.06**	**0.48 ± 0.22**	**0.75 ± 0.13**	**0.61 ± 0.14**	**0.51 ± 0.20**	**0.25 ± 0.12**	**0.64 ± 0.14**
**wav2vec 2.0 first transformer layer**	DT	0.75	0.70	0.80	0.81	0.73	0.53	0.75
	KNN	0.76	0.72	0.80	0.81	0.75	0.54	0.84
	LR	0.82	0.84	0.80	0.83	0.83	0.66	0.92
	NB	0.68	0.74	0.62	0.67	0.69	0.37	0.71
	RF	0.75	0.76	0.74	0.77	0.74	0.52	0.82
	SVM	0.80	0.82	0.78	0.81	0.81	0.62	0.90
	**mean ± std**	**0.76 ± 0.05**	**0.76 ± 0.06**	**0.76 ± 0.07**	**0.78 ± 0.06**	**0.76 ± 0.05**	**0.54 ± 0.10**	**0.82 ± 0.08**
**baselines**	DT	0.54	0.52	0.56	0.55	0.51	0.09	0.54
	KNN	0.54	0.52	0.56	0.56	0.53	0.08	0.61
	LR	0.61	0.64	0.58	0.67	0.62	0.23	0.64
	NB	0.54	0.64	0.44	0.55	0.59	0.07	0.56
	RF	0.56	0.54	0.58	0.59	0.55	0.13	0.65
	SVM	0.64	0.64	0.64	0.70	0.63	0.30	0.66
	**mean ± std**	**0.57 ± 0.04**	**0.58 ± 0.06**	**0.56 ± 0.07**	**0.60 ± 0.07**	**0.57 ± 0.05**	**0.15 ± 0.09**	**0.61 ± 0.05**

For both read text and monologue, the best performing Wav2Vec-based approaches demonstrated comparable results, with no significant performance differences observed between the two speech modes. However, the w2v2-LH was the poorest-performing representation. Baseline features underperformed compared to Wav2Vec embeddings, with performance drops exceeding 20 % across several metrics.

In contrast to the English dataset, results from PC-GITA exhibited lower variability across classifiers and more stable outcomes for higher-performing Wav2Vec representations. Further details on PCA-reduced Wav2Vec embeddings for the PC-GITA dataset are provided in Appendix D4 and Appendix D5.

#### 3.4.2 Optimized classifiers

For w2v1-FEA in the read text task, fine-tuning increased the mean accuracy from 0.76 to 0.80, with a maximum accuracy gain from 0.81 to 0.85. For w2v2–1T, fine-tuning increased the mean accuracy from 0.78 to 0.79, while the maximum accuracy remained unchanged at 0.82. For the dialogue task, fine-tuning raised the w2v1-FEA mean accuracy from 0.76 to 0.78, with an improvement in maximum accuracy from 0.81 to 0.83. For w2v2–1T, the mean accuracy remained at 0.76, while the maximum accuracy increased from 0.82 to 0.85.

**Fig. 4:**
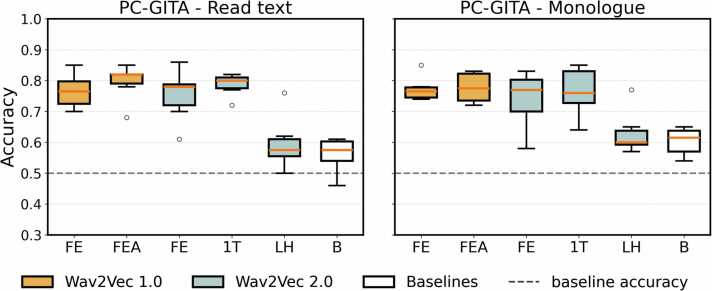
Distributions of accuracies across fine-tuned classifiers for the Spanish dataset.

Distributions of accuracies across fine-tuned classifiers for each representation can be found (Fig. 4). The best classification results obtained for each representation can be found in Table 9.

**Table 9:** Classification metrics of the best fine-tuned classifier for each feature extraction method.

**mode**	**representation**	**classifier**	**accuracy**	**sens.**	**spec.**	**AUC**	**F1**
**read text**	w2v1-FE	SVM	0.85	0.86	0.84	0.92	0.84
	w2v1-FEA	SVM	0.85	0.78	0.92	0.90	0.82
	w2v2-FE	SVM	0.86	0.86	0.86	0.88	0.86
	w2v2-1T	LR	0.82	0.80	0.84	0.88	0.82
	w2v2-LH	RF	0.76	0.74	0.78	0.86	0.75
	baselines	LR	0.61	0.62	0.60	0.68	0.62
**monologue**	w2v1-FE	SVM	0.85	0.86	0.84	0.91	0.85
	w2v1-FEA	RF	0.83	0.80	0.86	0.89	0.80
	w2v2-FE	RF	0.83	0.82	0.84	0.84	0.82
	w2v2-1T	SVM	0.85	0.88	0.82	0.91	0.85
	w2v2-LH	RF	0.77	0.72	0.82	0.85	0.76
	baselines	SVM	0.65	0.68	0.62	0.66	0.65

w2v2–1T: first transformer layer; w2v2-LH: last hidden layer; w2v2-FE: feature extraction; w2v1-FE: feature extraction; w2v1-FEA: feature extraction and aggregation

#### 3.4.3 Embeddings fusion

As shown in Table 10, combined embeddings (w2v1+w2v2) selected from the best-performing layers of Wav2Vec 1.0 and 2.0 did not lead to any significant performance improvements when compared with the best fine-tuned classifiers. In the case of the read text, accuracy (+0.01) and F1 score (+0.03) improved slightly.

**Table 10:** PC-GITA: classification performance (fine-tuned) using early fusion of Wav2Vec embeddings.

**mode**	**representation**	**classifier**	**accuracy**	**sens.**	**spec.**	**AUC**	**F1**
**read text**	w2v1-FEA	SVM	0.85	0.86	0.84	0.92	0.84
	w2v2-1T	LR	0.82	0.80	0.84	0.88	0.82
	v1 +v2	RF	0.86	0.90	0.82	0.92	0.87
**monologue**	w2v1-FEA	RF	0.83	0.80	0.86	0.89	0.80
	w2v2-1T	SVM	0.85	0.88	0.82	0.91	0.85
	v1 +v2	SVM	0.85	0.88	0.82	0.91	0.85

w2v1-FEA: feature extraction and aggregation; w2v2–1T: first transformer layer

### 3.5 Developed classification models using the sustained vowel /a/

#### 3.5.1 Classifiers used in TOPSIS

We further evaluated the performance of HC vs. PD classifiers on two vowel datasets, one from PC-GITA (Table 11) and the other from the U.S.-based dataset (Table 12) [26]. The w2v2-FE consistently achieved the best performance across both datasets. It yielded an accuracy of 0.69 and an AUC of 0.76 for the PC-GITA dataset, and an accuracy of 0.70 with an AUC of 0.75 for the US-based dataset. In addition, the w2v1-FE outperformed w2v1-FEA. Further comparisons, including PCA-reduced Wav2Vec embeddings for the vowel datasets, are detailed in Appendix D6 and Appendix D7.

**Table 11:** Classifier performance metrics for vowel dataset from the PC-GITA dataset, which included 50 HC and 50 PD samples in a balanced distribution.

	**classifier**	**accuracy**	**sens.**	**spec.**	**precision**	**F1**	**MCC**	**AUC**
**wav2vec 1.0 feature extractor**	DT	0.65	0.68	0.62	0.68	0.66	0.32	0.65
	KNN	0.64	0.66	0.62	0.65	0.64	0.29	0.66
	LR	0.65	0.64	0.66	0.67	0.64	0.32	0.71
	NB	0.69	0.66	0.72	0.72	0.67	0.40	0.72
	RF	0.70	0.64	0.76	0.73	0.66	0.42	0.73
	SVM	0.61	0.46	0.76	0.73	0.53	0.26	0.73
	**mean ± std**	**0.66 ± 0.03**	**0.62 ± 0.08**	**0.69 ± 0.07**	**0.70 ± 0.03**	**0.63 ± 0.05**	**0.34 ± 0.06**	**0.70 ± 0.04**
**wav2vec 1.0 feature extractor + aggregator**	DT	0.67	0.70	0.64	0.70	0.68	0.35	0.67
	KNN	0.54	0.60	0.48	0.54	0.56	0.07	0.57
	LR	0.60	0.58	0.62	0.64	0.59	0.21	0.71
	NB	0.57	0.44	0.70	0.67	0.51	0.17	0.62
	RF	0.67	0.66	0.68	0.71	0.67	0.35	0.70
	SVM	0.58	0.54	0.62	0.60	0.55	0.17	0.66
	**mean ± std**	**0.61 ± 0.05**	**0.59 ± 0.09**	**0.62 ± 0.08**	**0.64 ± 0.07**	**0.59 ± 0.07**	**0.22 ± 0.11**	**0.66 ± 0.05**
**wav2vec 2.0 feature extractor**	DT	0.58	0.64	0.52	0.54	0.57	0.18	0.58
	KNN	0.74	0.74	0.74	0.76	0.74	0.49	0.79
	LR	0.71	0.76	0.66	0.71	0.73	0.43	0.83
	NB	0.66	0.78	0.54	0.63	0.69	0.35	0.73
	RF	0.75	0.78	0.72	0.75	0.76	0.51	0.83
	SVM	0.70	0.70	0.70	0.72	0.70	0.40	0.79
	**mean ± std**	**0.69 ± 0.06**	**0.73 ± 0.05**	**0.65 ± 0.09**	**0.69 ± 0.09**	**0.70 ± 0.07**	**0.39 ± 0.12**	**0.76 ± 0.09**
**wav2vec 2.0 last hidden layer**	DT	0.49	0.44	0.54	0.50	0.46	−0.02	0.49
	KNN	0.68	0.72	0.64	0.71	0.69	0.38	0.68
	LR	0.59	0.48	0.70	0.61	0.53	0.19	0.64
	NB	0.62	0.68	0.56	0.62	0.64	0.25	0.65
	RF	0.70	0.76	0.64	0.68	0.71	0.41	0.76
	SVM	0.56	0.36	0.76	0.62	0.43	0.14	0.42
	**mean ± std**	**0.61 ± 0.08**	**0.57 ± 0.17**	**0.64 ± 0.08**	**0.62 ± 0.07**	**0.58 ± 0.12**	**0.23 ± 0.16**	**0.61 ± 0.13**
**wav2vec 2.0 first transformer layer**	DT	0.64	0.68	0.60	0.65	0.65	0.29	0.64
	KNN	0.73	0.76	0.70	0.72	0.74	0.47	0.79
	LR	0.68	0.66	0.70	0.71	0.67	0.38	0.76
	NB	0.66	0.76	0.56	0.65	0.69	0.35	0.71
	RF	0.69	0.70	0.68	0.71	0.70	0.39	0.79
	SVM	0.70	0.68	0.72	0.71	0.69	0.41	0.74
	**mean ± std**	**0.68 ± 0.03**	**0.71 ± 0.04**	**0.66 ± 0.06**	**0.69 ± 0.03**	**0.69 ± 0.03**	**0.38 ± 0.06**	**0.74 ± 0.06**
**baselines**	DT	0.56	0.58	0.54	0.57	0.56	0.12	0.56
	KNN	0.47	0.40	0.54	0.49	0.42	−0.07	0.52
	LR	0.55	0.42	0.68	0.49	0.44	0.10	0.60
	NB	0.56	0.32	0.80	0.69	0.40	0.16	0.56
	RF	0.64	0.68	0.60	0.66	0.65	0.30	0.71
	SVM	0.52	0.22	0.82	0.55	0.29	0.07	0.31
	**mean ± std**	**0.55 ± 0.06**	**0.44 ± 0.17**	**0.66 ± 0.12**	**0.58 ± 0.09**	**0.46 ± 0.13**	**0.11 ± 0.12**	**0.55 ± 0.13**

**Table 12:** Classifier performance metrics for vowel dataset from the U.S.-based dataset, which included 41 HC and 40 PD samples in a balanced distribution.

	**classifier**	**accuracy**	**sens.**	**spec.**	**precision**	**F1**	**MCC**	**AUC**
**wav2vec 1.0 feature extractor**	DT	0.65	0.70	0.61	0.64	0.66	0.32	0.65
	KNN	0.54	0.68	0.42	0.58	0.58	0.11	0.62
	LR	0.63	0.65	0.62	0.64	0.63	0.28	0.68
	NB	0.61	0.50	0.71	0.62	0.54	0.22	0.64
	RF	0.69	0.65	0.74	0.75	0.66	0.42	0.77
	SVM	0.63	0.63	0.64	0.66	0.61	0.29	0.39
	**mean ± std**	**0.63 ± 0.05**	**0.63 ± 0.07**	**0.62 ± 0.11**	**0.65 ± 0.06**	**0.61 ± 0.05**	**0.27 ± 0.10**	**0.62 ± 0.13**
**wav2vec 1.0 feature extractor + aggregator**	DT	0.57	0.68	0.46	0.59	0.60	0.14	0.57
	KNN	0.65	0.68	0.63	0.70	0.66	0.33	0.69
	LR	0.62	0.65	0.59	0.65	0.62	0.26	0.65
	NB	0.64	0.58	0.71	0.59	0.58	0.30	0.67
	RF	0.56	0.55	0.57	0.50	0.51	0.13	0.66
	SVM	0.62	0.65	0.58	0.62	0.62	0.24	0.69
	**mean ± std**	**0.61 ± 0.04**	**0.63 ± 0.05**	**0.59 ± 0.08**	**0.61 ± 0.07**	**0.60 ± 0.05**	**0.23 ± 0.08**	**0.65 ± 0.05**
**wav2vec 2.0 feature extractor**	DT	0.60	0.68	0.53	0.59	0.62	0.22	0.60
	KNN	0.65	0.73	0.58	0.68	0.66	0.34	0.67
	LR	0.71	0.75	0.68	0.72	0.72	0.45	0.80
	NB	0.74	0.75	0.73	0.74	0.74	0.50	0.78
	RF	0.75	0.78	0.73	0.76	0.76	0.51	0.82
	SVM	0.73	0.75	0.70	0.74	0.72	0.48	0.83
	**mean ± std**	**0.70 ± 0.06**	**0.74 ± 0.03**	**0.66 ± 0.08**	**0.70 ± 0.06**	**0.70 ± 0.05**	**0.42 ± 0.11**	**0.75 ± 0.09**
**wav2vec 2.0 last hidden layer**	DT	0.64	0.65	0.64	0.69	0.64	0.29	0.64
	KNN	0.64	0.75	0.53	0.62	0.67	0.29	0.71
	LR	0.62	0.40	0.83	0.59	0.46	0.24	0.72
	NB	0.60	0.48	0.73	0.68	0.53	0.23	0.73
	RF	0.65	0.65	0.65	0.68	0.63	0.33	0.78
	SVM	0.51	0.00	1.00	0.00	0.00	0.00	0.29
	**mean ± std**	**0.61 ± 0.06**	**0.49 ± 0.27**	**0.73 ± 0.17**	**0.54 ± 0.27**	**0.49 ± 0.25**	**0.23 ± 0.12**	**0.64 ± 0.18**
**wav2vec 2.0 first transformer layer**	DT	0.61	0.58	0.64	0.58	0.56	0.22	0.61
	KNN	0.67	0.68	0.66	0.71	0.65	0.37	0.72
	LR	0.68	0.65	0.70	0.75	0.66	0.39	0.73
	NB	0.76	0.75	0.78	0.78	0.76	0.54	0.78
	RF	0.73	0.73	0.73	0.73	0.72	0.46	0.80
	SVM	0.62	0.65	0.59	0.65	0.63	0.27	0.73
	**mean ± std**	**0.68 ± 0.06**	**0.67 ± 0.06**	**0.68 ± 0.07**	**0.70 ± 0.07**	**0.66 ± 0.07**	**0.38 ± 0.12**	**0.73 ± 0.07**
**baselines**	DT	0.54	0.55	0.53	0.61	0.53	0.11	0.54
	KNN	0.61	0.50	0.72	0.68	0.55	0.24	0.64
	LR	0.64	0.50	0.78	0.74	0.58	0.32	0.73
	NB	0.63	0.43	0.83	0.79	0.53	0.31	0.67
	RF	0.57	0.55	0.59	0.60	0.55	0.16	0.65
	SVM	0.65	0.40	0.91	0.78	0.50	0.36	0.73
	**mean ± std**	**0.61 ± 0.04**	**0.49 ± 0.06**	**0.73 ± 0.14**	**0.70 ± 0.09**	**0.54 ± 0.02**	**0.25 ± 0.10**	**0.66 ± 0.07**

#### 3.5.2 Optimized classifiers

For w2v2-FE on the PC-GITA vowels, fine-tuning did not change the mean accuracy, which remained at 0.69, nor the maximum accuracy, which stayed at 0.75. For w2v2–1T, fine-tuning slightly decreased the mean accuracy from 0.68 to 0.67. On the vowels dataset [26], fine-tuning reduced the mean accuracy of w2v2-FE from 0.70 to 0.67, while for w2v2–1T, fine-tuning improved the mean accuracy from 0.68 to 0.70. Overall, fine-tuned models generally did not perform better than their non-fine-tuned counterparts.

**Fig. 5:**
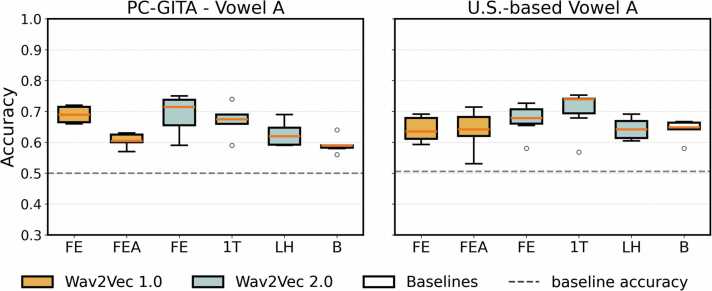
Distributions of accuracies across fine-tuned classifiers for the isolated vowels.

Distributions of accuracies across fine-tuned classifiers for each representation can be found (Fig. 5). The best classification results obtained for each representation can be found in Table 13.

**Table 13:** Classification metrics of the best fine-tuned classifier for each feature extraction method.

**mode**	**representation**	**classifier**	**accuracy**	**sens.**	**spec.**	**AUC**	**F1**
**PC-GITA** **vowels**	w2v1-FE	SVM	0.72	0.72	0.72	0.78	0.72
	w2v1-FEA	NB	0.63	0.54	0.72	0.68	0.59
	w2v2-FE	RF	0.75	0.78	0.72	0.85	0.75
	w2v2-1T	KNN	0.74	0.78	0.70	0.79	0.75
	w2v2-LH	RF	0.69	0.76	0.62	0.69	0.71
	baselines	SVM	0.64	0.50	0.78	0.62	0.58
**vowels /a/** [26]	w2v1-FE	RF	0.69	0.68	0.71	0.74	0.68
	w2v1-FEA	KNN	0.71	0.75	0.68	0.76	0.73
	w2v2-FE	NB	0.73	0.70	0.76	0.78	0.71
	w2v2-1T	SVM	0.75	0.70	0.80	0.73	0.74
	w2v2-LH	DT	0.69	0.63	0.76	0.68	0.67
	baselines	LR	0.67	0.53	0.80	0.71	0.61

w2v2–1T: first transformer layer; w2v2-LH: last hidden layer; w2v2-FE: feature extraction; w2v1-FE: feature extraction; w2v1-FEA: feature extraction and aggregation

#### 3.5.3 Embeddings fusion

As shown in Table 14, combined embeddings (w2v1+w2v2) selected from the best-performing layers of Wav2Vec 1.0 and 2.0 improved metrics for both datasets. Interestingly, for PC-GITA, we observed a significant improvement, reaching an accuracy of 0.80 (+0.05), which was the highest accuracy reported for this dataset across all presented experiments.

**Table 14:** Isolated vowels: classification performance (fine-tuned) using early fusion of Wav2Vec embeddings.

**mode**	**representation**	**classifier**	**accuracy**	**sens.**	**spec.**	**AUC**	**F1**
**PC-GITA** **vowels**	w2v1-FE	SVM	0.72	0.72	0.72	0.78	0.72
	w2v2-FE	RF	0.75	0.78	0.72	0.85	0.75
	v1 +v2	RF	0.80	0.84	0.76	0.86	0.81
**vowels /a/** [26]	w2v1-FE	RF	0.69	0.68	0.71	0.74	0.68
	w2v2-FE	NB	0.73	0.70	0.76	0.78	0.71
	v1 +v2	RF	0.75	0.75	0.75	0.82	0.75

w2v1-FE: feature extraction; w2v2-FE: feature extraction

## 4 Discussion

This study demonstrated the superior performance of Wav2Vec 2.0 across datasets and speech modes, while also showing that Wav2Vec 1.0 remained highly competitive, delivering comparable results and, in certain fine-tuned cases, even outperforming Wav2Vec 2.0. By employing the TOPSIS multicriteria decision analysis, we systematically ranked the different speech representations based on average performance metrics across classifiers. Our findings emphasize the importance of carefully selecting an appropriate layer when applying Wav2Vec-based approaches for PD detection, as performance might vary depending on the layer utilized. Wav2Vec 1.0 was the fastest in terms of runtime. We used TOPSIS as the primary method for systematic evaluation to ensure a fair comparison. We believe that maintaining default hyperparameters within TOPSIS is the fairest way to compare different feature representations, i.e. any change in ranking should stem from the embeddings themselves rather than classifier tuning. Finally, a post-hoc fine-tuning analysis illustrated the potential upper bound of performance without interfering with the main benchmarking strategy. Across all datasets, the TOPSIS ranking based on average accuracy scores was strongly consistent with the results obtained from optimized classifiers.

An important finding was that w2v1-large trained exclusively on English remained competitive with the multilingual w2v2 XLSR-53 and, in some cases, even displayed greater performance, as in the Spanish PC-GITA dataset, suggesting that its CNN based feature extractor captures pathological speech cues independently of language. Hyperparameter optimization further demonstrated that w2v1 can outperform XLSR-53, particularly on the MDVR-KCL English dataset. By combining selected layers from both w2v1 and w2v2 in an early fusion approach, we developed composite embeddings that improved diagnostic accuracy in vowel-based classification (+0.05 in accuracy).

Fine tuning enhanced performance for connected speech tasks in MDVR-KCL and PC-GITA but produced limited or negative effects on vowel tasks; in some cases, such as when NB w2v2–1T was applied to vowel classification, a non fine tuned model performed better than a fine tuned one. After fine tuning, spontaneous dialogue in MDVR-KCL using w2v1-FE achieved performance equivalent to that of the read text task. This indicated that effective detection of PD may be possible without diarization [38], i.e. without partitioning an audio stream into homogeneous segments based on speaker identity and processing the remaining content as monologues. In general, fine tuned models did not outperform their non fine tuned counterparts in vowel-based tasks. Moreover, some classifiers used in TOPSIS exhibited AUC values below 0.5, indicating systematic prediction errors or label inversions rather than true performance. We retained these low accuracy outcomes as penalties for ranking, though careful parameter tuning may prevent such results. Table 15 indicates the performance analysis under various state-of-the-art techniques.

**Table 15:** Accuracy analysis under various state-of-the-art voice and speech models.

**dataset**	**task**	**study**	**SFM used**	**method**	**classifier**	**accuracy (std)**
English MDVR-KCL	read text	*Cesare et al., (2024) [38]	No	MFCC + GTCC	NN	92 %
		**proposed	Yes	w2v1-FEA	SVM	86 %
			Yes	w2v2-1T	SVM	86 %
	dialogue	*Cesare et al., (2024) [38]	No	MFCC + GTCC	SVM	90 %
		**proposed	Yes	w2v1-FE	DT	90 %
			Yes	w2v2-1T	NB	80 %
Spanish PC-GITA	read text	Sheikh, 2024 [17]	Yes	w2v2-XLSR-4T	NN	85 %
		Reszka et al., 2024 [39]	Yes	w2v2 + SGMSE	NN	82 % (13.0)
		Sheikh and Kodrasi, 2024 [18]	Yes	w2v2-XLSR-1T	NN	77 % (14.9)
		Purohit et al., 2025 [14]	Yes	w2v2-base-10T	NN	84 % (5.6)
			Yes	w2v2-XLSR-16T	NN	84 % (8.3)
			Yes	Whisper	NN	81 % (8.6)
		**proposed**	Yes	w2v1-FEA	SVM	85 %
			Yes	w2v2-FE	SVM	86 %
	monologue	Escobar-Grisales et al., 2024 [40]	Yes	w2v2-base-4T	NN	82 % (4.0)
		Sheikh and Kodrasi, 2024 [18]	Yes	w2v2-XLSR-4T	NN	75 % (15.8)
		**proposed**	Yes	w2v1-FE	SVM	85 %
			Yes	w2v2-1T	SVM	85 %
	vowels	Karan et al., 2021 [41]	No	HCC	NN	91 %
		Hires et al., 2023 [42]	No	spectrogram	CNN	91 %
		**proposed**	Yes	w2v1-FE + w2v2-FE	RF	80 %
vowels [26]	vowels	Iyer et al., 2023 [26]	No	MFCC	RF	NA*** (AUC = 0.73)
			No	spectrogram	CNN	NA*** (AUC = 0.97)
		**proposed**	Yes	w2v1-FE + w2v2-FE	RF	75 % (AUC = 0.82)

MFCC: Mel-frequency cepstral coefficients; GTCC: Gammatone frequency cepstral coefficients; HCC: Hilbert cepstral coefficients; w2v2-base-4T: 4-th layer of the wav2vec 2.0 base model; *Data was partitioned in order to increase the dataset size; **The evaluation was conducted on full-length audio recordings; SGMSE: Score-based generative model for speech enhancement; ***NA: accuracy not available

In a recent study from Cesare et al. [38], accuracy greater than 90 % and improved sensitivity for both read text and spontaneous dialogue using non-DL cepstral features were observed when classifying the English dataset. While these results surpassed the classification metrics observed in this study, the AUC and other additional metrics were not reported. The performance presented in our previous study [23], which focused only on the read text task using Wav2Vec 1.0, aligned with our current observations, as expected. In addition, this study offered a comparison of several classifiers and provided performance data across metrics, including accuracy, sensitivity, specificity, F1 score, and MCC. Importantly, we observed an improvement of up to 15 % in the AUC.

For the PC-GITA dataset, recent studies from 2024 have identified Wav2Vec 2.0 as a top-tier-performing foundational speech model. One such study [10] utilized the base model of Wav2Vec 2.0 and reported an average accuracy of 80 % and an AUC of 0.8, results that are consistent with the findings presented in this study. Building on this, significant improvements were demonstrated in the monologue task when frame-wise information extracted from the Wav2Vec 2.0 representations was incorporated [40]. Further advancements in PD detection include the application of diffusion-based conditional generative models for speech enhancement [39]. This method separated clean signals and residue signals from the original recordings, leading to enhanced classifier performance for dysarthric speech. When paired with the Wav2Vec 2.0 representation and a multilayer perceptron classifier, an AUC of 0.83, an accuracy of 0.79, a sensitivity of 0.74, and a specificity of 84 % were achieved [39]. These findings align closely with our observations.

For the sustained vowel /a/ datasets, prior studies have demonstrated that specific algorithms and approaches can outperform wav2vec-based methods in certain aspects and that wav2vec is likely to be more suitable for complex speech tasks over simple tasks like sustained vowel classification. Hires et al. achieved an exceptional performance on the PC-GITA dataset using a CNN model, reporting an AUC exceeding 0.9 [42]. Our findings align more closely with results from their study obtained using a comprehensive set of traditional, non-DL features, which achieved an average AUC of 0.88, approximately 2 % higher than the best AUC observed with w2v1+w2v2 and RF classifier in this study. On the U.S.-based vowels dataset, our SVM w2v2-based approach achieved an accuracy of 0.75, slightly outperforming results seen across the four feature sets used by Iyer et al., where the reported accuracy was 0.73 [26]. Additionally, their research highlighted a transfer-learned CNN solution, which achieved a performance comparable to the CNN used by Hires et al. Nevertheless, Wav2Vec embeddings proved to be relatively competitive with other state-of-the-art methods, offering a viable alternative for sustained vowel tasks, particularly when considering their broader applicability across diverse speech modes and disorders.

In the context of [18], our findings are highly comparable, particularly regarding PC-GITA. The referenced study reported an average accuracy of 77 % for non-spontaneous speech and 75 % for spontaneous speech, focusing exclusively on embeddings extracted from the w2v2-XLSR-1T. In this study, we extended these findings by analyzing outputs from additional layers of the Wav2Vec 2.0 architecture. Furthermore, as noted in [18], the final layers of Wav2Vec 2.0 are more effective for tasks focused on phonetic or non-paralinguistic content. In contrast, earlier layers are better suited for capturing paralinguistic and prosodic features, such as rhythm and intonation. This is consistent with our findings, i.e. that features extracted from lower layers outperformed those from higher layers, suggesting that lower layers more effectively capture acoustic characteristics that distinguish PD from HC.

Importantly, our results also demonstrated consistency with the previously reported compensatory patterns between spontaneous and non-spontaneous speech modes in Wav2Vec 2.0-based models. These models effectively extract additional cues in spontaneous speech that are less accessible in non-spontaneous speech, as highlighted in [18]. Interestingly, we observed a similar trend of compensation with Wav2Vec 1.0, extending this behavior across both architectures.

Across speech modes, classification on the English dataset using PCA, which was applied in order to reduce the dimensionality of the wav2vec representations, resulted in a significant drop in performance. Similar or slightly reduced performance was also observed for PC-GITA and vowel datasets compared to classification using the full feature set. This indicated that while dimensionality reduction can simplify data processing, it may lead to the loss of critical information for deep embeddings.

Wav2Vec 1.0 was observed to provide a good balance between speed and accuracy. The results obtained in this study should encourage the use of w2v1 in the context of lightweight solutions, such as web applications. We believe w2v1 could be integrated into tools like those described in [43]. With a user-friendly interface (e.g., Streamlit), the system could allow direct audio uploads, eliminating the need for manual feature extraction, and enabling server-side processing. Such applications would be particularly suitable for early PD detection and predicting clinical scores from speech [15] in a cost-effective manner.

This study has certain limitations. The input performance metrics used for the TOPSIS rankings were highly inter-dependent and correlated, and we did not conduct sensitivity analyses or experiment with weighting different metrics. Another current limitation of this work is the absence of advanced classifiers such as NN or other DL approaches. Additionally, we did not explore any fine-tuning approaches of the Wav2Vec architectures. Fine-tuning, in general, is highly computationally demanding, and should be pursued in a future study with greater resources and scope. Notably, fine-tuning Wav2Vec 2.0 for dysarthria has been attempted by [14], [16], [17], setting a precedent for future work in this domain. Moreover, approaches such as classifier combination and attention-based feature fusion, as presented in [4], could be further explored to establish optimal layer utilization strategies. While such strategies have been applied to tasks like emotion detection, they have yet to be explored in the context of dysarthric speech.

## 5 Conclusion

This study established Wav2Vec 2.0 as the top-performing speech representation technique for PD detection across connected speech and vowel-based classifications. Its superiority highlighted its utility in identifying PD-related speech characteristics. However, we also highlight that Wav2Vec 1.0 should not be overlooked. Despite its simpler, non-transformer architecture, it demonstrated competitive performance and remained a valuable alternative, particularly when rapid computation is prioritized over marginal performance gains. Its faster processing times make it a promising option for applications like federated learning, where computational efficiency, data anonymization, compression, and secure distributed analysis are essential. Our findings extend existing theories on disorder classification using Wav2Vec by offering new insights into the behavior of different speech modes. As part of our future work, we plan to extend our research by including advanced classifiers such as NNs and other DL approaches that can operate directly on full-length embeddings without the need for pooling or aggregation.

## Ethics statements and data availability

The datasets used in this study are publicly available, with one dataset, PC-GITA, being available for download upon request from their authors. The PC-GITA dataset is available upon request from Juan Rafael Orozco-Arroyave affiliated with Universidad de Antioquia UdeA. The study complied with the Helsinki Declaration and was approved by the Ethics Committee of Clinica Noel in Medellín, Colombia. A written informed consent was signed by each participant.

## Role of the funding source

The authors declare that the study sponsors had no role in the design of the study, the collection, analysis, and interpretation of data, the writing of the manuscript, or the decision to submit the manuscript for publication. All sources of funding are duly acknowledged, and no external influence has affected the integrity or independence of this research.

## Author statement

All authors have reviewed and approved the final version of this revised manuscript.

## CRediT authorship contribution statement

**Ondrej Klempir:** Writing – review & editing, Writing – original draft, Visualization, Validation, Software, Methodology, Investigation, Formal analysis, Data curation, Conceptualization. **Adela Skryjova:** Writing – review & editing, Visualization, Software, Data curation. **Ales Tichopad:** Writing – review & editing, Methodology, Formal analysis. **Radim Krupicka:** Writing – review & editing, Supervision, Project administration, Funding acquisition, Formal analysis.

## Declaration of generative AI and AI-assisted technologies in the writing process

During the preparation of this work the authors used ChatGPT to improve the readability and language of the manuscript. After using this tool, the authors reviewed and edited the content as needed and take full responsibility for the content of the published article. After refining the manuscript's language with ChatGPT, it was further reviewed and enhanced by a native speaker to ensure clarity and accuracy.

## Declaration of Competing Interest

None Declared

## Appendix B Baseline features

The following audio frame features (A-F) were extracted for each audio signal:

A. Energy Entropy
  (1) $${\mathrm{EE}}_{t}=\;-\sum_{s=1}^{S}s\;*\;\log_{2}s,$$
  the time domain signal for frame t is split into 10 short blocks. S is the number of short blocks, s corresponds to the energy of a short block.
B. Energy
  (2) $$E_{t}=\;\sqrt{\frac{1}{N}\sum_{n=1}^{N}{\left|x[n]\right|}^{2}},$$
  the root-mean-square (RMS) level of a signal reflects the signal energy.
C. Zero Crossing Rate
  (3) $$Z_{t}=\;\frac{1}{2}\sum_{n=1}^{N}\left|\mathrm{sign}\left(x[n]\right)-\mathrm{sign}(x[n-1])\right|,$$
  a measure of the noisiness of the signal. $x\left[n\right]\;$is the time domain signal for frame t.
D. Spectral Rolloff
  The frequency such that,
  (4) $$\sum_{n=1}^{R_{t}}M_{t}\left[n\right]=0.85\sum_{n=1}^{N}M_{t}\left[n\right],$$
  a measure of the skewness of the spectral shape. $M_{t}[n]$ is the magnitude of the Fourier transform at frame t and frequency bin n.
E. Spectral Centroid
  (5) $$C_{t}=\frac{\;\sum_{n=1}^{N}M_{t}\left[n\right]*n}{\sum_{n=1}^{N}M_{t}[n]},$$
  the center of gravity of the magnitude spectrum of a short-time Fourier transform.
F. Spectral Flux

(6) $$F_{t}=\sum_{n=1}^{N}(N_{t}\left[n\right]-{N_{t-1}\left[n\right])}^{2},$$

a measure of the amount of local spectral change. $N_{t}\left[n\right]$ and $N_{t-1}\left[n\right]$ are the normalized magnitude of the Fourier transform at frames t and t−1.

## Appendix C TOPSIS

1. Data gathering: We created a decision matrix where each row represented a different method and each column represented a metric. We then did this for each speech mode.
2. Data normalization: We normalized the decision matrix so that the metrics could be compared on the same scale. Vector normalization was implemented by dividing each element by the square root of the sum of squares of all elements in its column (Euclidean norm). We used this formula for normalization:
  $$r_{\mathrm{ij}}\;=\;\frac{x_{\mathrm{ij}}}{\sqrt{\sum_{i=1}^{m}x_{\mathrm{ij}}^{2}}}$$
  Where $r_{\mathrm{ij}}\;$ is the normalized value for method i and criterion j, and $x_{\mathrm{ij}}$ is the original value.
3. Assigning weights to metrics and calculation of weighted normalized matrix: We assumed equal importance for each metric (the sum of all weights equals 1). The process was then followed by multiplying each element of the normalized decision matrix by the weight of the corresponding metric:
  $$v_{\mathrm{ij}}\;=\;w_{\mathrm{ij}}\cdot r_{\mathrm{ij}}$$
4. Ideal and Anti-Ideal Solutions: For each metric, we identified the best and worst values from the weighted normalized matrix. The best values formed the “ideal best” and the worst values formed the “ideal worst” solution. Note that “best” may typically mean highest or lowest depending on whether the metric is a benefit or a cost criterion—in this study, all machine learning metrics are to be maximized.
5. Calculate the separation measures: For each alternative methods, calculate the Euclidean distance from the weighted normalized scores to both the ideal best and ideal worst:
  $$D_{i}^{+}=\;\sqrt{\sum_{j=1}^{n}{\left(v_{\mathrm{ij}}-A_{j}^{+}\right)}^{2}}$$
  $$D_{i}^{-}=\;\sqrt{\sum_{j=1}^{n}{\left(v_{\mathrm{ij}}-A_{j}^{-}\right)}^{2}}$$
6. Calculate relative closeness: For each method, calculate the relative closeness to the ideal solution using the separation from the ideal best and ideal worst. This is done by dividing the separation from the ideal worst by the sum of the separation from the ideal best and ideal worst:
  $$C_{i}^{*}\;=\;\frac{D_{i}^{-}}{D_{i}^{+}+\;D_{i}^{-}\;}$$
7. Rank the alternatives: The method with the highest $C_{i}^{*}$ is preferred. A higher relative closeness indicates a better performance according to the specified metrics and weights.
