# Supplementary material for: Ranking pre-trained speech embeddings in Parkinson's disease detection: Does Wav2Vec 2.0 outperform its 1.0 version across speech modes and languages?
Source: Comput Struct Biotechnol J. 2025 Jun 7;27:2584–601. doi: 10.1016/j.csbj.2025.06.022 (PMC12206144; doi:10.1016/j.csbj.2025.06.022)
Supplement: Supplementary file 1 — Supplementary material [file mmc1.docx]

# **Appendix D. Supplementary tables**

**Table D1**. MCDA TOPSIS.

| **dataset** | **mode** | **accuracy** | **sens.** | **spec.** | **precision** | **F1** | **MCC** | **AUC** | **TOPSIS** | **rank** | **method** |
| --- | --- | --- | --- | --- | --- | --- | --- | --- | --- | --- | --- |
| English [24] | read text | 0.67 | 0.36 | 0.90 | 0.43 | 0.37 | 0.28 | 0.72 | 0.41 | 4 | w2v1-FE |
|  |  | 0.75 | 0.48 | 0.93 | 0.63 | 0.53 | 0.44 | 0.75 | 0.85 | 2 | w2v1-FEA |
|  |  | 0.63 | 0.33 | 0.85 | 0.32 | 0.30 | 0.19 | 0.67 | 0.18 | 5 | w2v2-FE |
|  |  | 0.59 | 0.28 | 0.83 | 0.35 | 0.30 | 0.13 | 0.55 | 0.07 | 6 | w2v2-LH |
|  |  | 0.71 | 0.58 | 0.81 | 0.58 | 0.54 | 0.41 | 0.84 | 0.88 | 1 | w2v2-1T |
|  |  | 0.64 | 0.49 | 0.75 | 0.51 | 0.47 | 0.25 | 0.65 | 0.51 | 3 | baselines |
|  | dialogue | 0.68 | 0.41 | 0.89 | 0.38 | 0.38 | 0.30 | 0.71 | 0.66 | 4 | w2v1-FE |
|  |  | 0.66 | 0.52 | 0.81 | 0.47 | 0.46 | 0.32 | 0.69 | 0.79 | 3 | w2v1-FEA |
|  |  | 0.60 | 0.36 | 0.80 | 0.29 | 0.29 | 0.16 | 0.65 | 0.43 | 5 | w2v2-FE |
|  |  | 0.53 | 0.18 | 0.77 | 0.19 | 0.17 | -0.06 | 0.45 | 0.02 | 6 | w2v2-LH |
|  |  | 0.65 | 0.59 | 0.73 | 0.48 | 0.50 | 0.31 | 0.71 | 0.81 | 2 | w2v2-1T |
|  |  | 0.69 | 0.57 | 0.80 | 0.60 | 0.54 | 0.39 | 0.73 | 0.95 | 1 | baselines |
| PC-GITA [25] | read text | 0.74 | 0.66 | 0.82 | 0.81 | 0.70 | 0.51 | 0.73 | 0.81 | 3 | w2v1-FE |
|  |  | 0.76 | 0.71 | 0.80 | 0.80 | 0.73 | 0.53 | 0.83 | 0.89 | 2 | w2v1-FEA |
|  |  | 0.70 | 0.72 | 0.68 | 0.73 | 0.70 | 0.42 | 0.76 | 0.68 | 4 | w2v2-FE |
|  |  | 0.55 | 0.46 | 0.64 | 0.53 | 0.47 | 0.10 | 0.59 | 0.10 | 6 | w2v2-LH |
|  |  | 0.78 | 0.78 | 0.77 | 0.81 | 0.77 | 0.57 | 0.82 | 0.96 | 1 | w2v2-1T |
|  |  | 0.57 | 0.62 | 0.53 | 0.57 | 0.58 | 0.15 | 0.59 | 0.20 | 5 | baselines |
|  | monologue | 0.73 | 0.66 | 0.79 | 0.77 | 0.69 | 0.47 | 0.74 | 0.77 | 4 | w2v1-FE |
|  |  | 0.76 | 0.71 | 0.81 | 0.80 | 0.74 | 0.54 | 0.82 | 0.93 | 2 | w2v1-FEA |
|  |  | 0.74 | 0.77 | 0.71 | 0.75 | 0.75 | 0.51 | 0.80 | 0.87 | 3 | w2v2-FE |
|  |  | 0.62 | 0.48 | 0.75 | 0.61 | 0.51 | 0.25 | 0.64 | 0.27 | 5 | w2v2-LH |
|  |  | 0.76 | 0.76 | 0.76 | 0.78 | 0.76 | 0.54 | 0.82 | 0.94 | 1 | w2v2-1T |
|  |  | 0.57 | 0.58 | 0.56 | 0.60 | 0.57 | 0.15 | 0.61 | 0.13 | 6 | baselines |
|  | vowels | 0.66 | 0.62 | 0.69 | 0.70 | 0.63 | 0.34 | 0.70 | 0.75 | 3 | w2v1-FE |
|  |  | 0.61 | 0.59 | 0.62 | 0.64 | 0.59 | 0.22 | 0.66 | 0.43 | 4 | w2v1-FEA |
|  |  | 0.69 | 0.73 | 0.65 | 0.69 | 0.70 | 0.39 | 0.76 | 0.95 | 1 | w2v2-FE |
|  |  | 0.61 | 0.57 | 0.64 | 0.62 | 0.58 | 0.23 | 0.61 | 0.41 | 5 | w2v2-LH |
|  |  | 0.68 | 0.71 | 0.66 | 0.69 | 0.69 | 0.38 | 0.74 | 0.93 | 2 | w2v2-1T |
|  |  | 0.55 | 0.44 | 0.66 | 0.58 | 0.46 | 0.11 | 0.55 | 0.05 | 6 | baselines |
| vowels /a/  [26] | vowels | 0.63 | 0.63 | 0.62 | 0.65 | 0.61 | 0.27 | 0.62 | 0.40 | 3 | w2v1-FE |
|  |  | 0.61 | 0.63 | 0.59 | 0.61 | 0.60 | 0.23 | 0.65 | 0.30 | 5 | w2v1-FEA |
|  |  | 0.70 | 0.74 | 0.66 | 0.70 | 0.70 | 0.42 | 0.75 | 0.89 | 1 | w2v2-FE |
|  |  | 0.61 | 0.49 | 0.73 | 0.54 | 0.49 | 0.23 | 0.64 | 0.20 | 6 | w2v2-LH |
|  |  | 0.68 | 0.67 | 0.68 | 0.70 | 0.66 | 0.38 | 0.73 | 0.78 | 2 | w2v2-1T |
|  |  | 0.61 | 0.49 | 0.73 | 0.70 | 0.54 | 0.25 | 0.66 | 0.30 | 4 | baselines |

w2v2-1T: first transformer layer; w2v2-LH: last hidden layer; w2v2-FE: feature extraction; w2v1-FE: feature extraction; w2v1-FEA: feature extraction and aggregation

**Table D2.** Classifier performance metrics for read text from the English dataset, which included 21 HC and 16 PD samples in an imbalanced distribution. Results reported for the 30 PCA components.

|  | **classifier** | **accuracy** | **sens.** | **spec.** | **precision** | **F1** | **MCC** | **AUC** |
| --- | --- | --- | --- | --- | --- | --- | --- | --- |
| **wav2vec 1.0 feature extractor** | DT | 0.54 | 0.35 | 0.65 | 0.25 | 0.28 | -0.02 | 0.50 |
|  | KNN | 0.73 | 0.35 | 1.00 | 0.50 | 0.40 | 0.37 | 0.90 |
|  | LR | 0.58 | 0.00 | 1.00 | 0.00 | 0.00 | 0.00 | 0.85 |
|  | NB | 0.53 | 0.45 | 0.58 | 0.47 | 0.43 | 0.07 | 0.60 |
|  | RF | 0.56 | 0.30 | 0.77 | 0.30 | 0.28 | 0.07 | 0.55 |
|  | SVM | 0.58 | 0.00 | 1.00 | 0.00 | 0.00 | 0.00 | 0.33 |
|  | **mean ± std** | **0.59 ± 0.07** | **0.24 ± 0.19** | **0.83 ± 0.19** | **0.25 ± 0.22** | **0.23 ± 0.19** | **0.08 ± 0.15** | **0.62 ± 0.22** |
| **wav2vec 1.0 feature extractor + aggregator** | DT | 0.40 | 0.15 | 0.57 | 0.20 | 0.17 | -0.30 | 0.36 |
|  | KNN | 0.71 | 0.30 | 1.00 | 0.50 | 0.37 | 0.33 | 0.73 |
|  | LR | 0.74 | 0.45 | 0.95 | 0.60 | 0.50 | 0.42 | 0.73 |
|  | NB | 0.67 | 0.40 | 0.82 | 0.50 | 0.43 | 0.23 | 0.66 |
|  | RF | 0.57 | 0.30 | 0.75 | 0.33 | 0.28 | 0.06 | 0.48 |
|  | SVM | 0.73 | 0.35 | 1.00 | 0.50 | 0.40 | 0.37 | 0.75 |
|  | **mean ± std** | **0.64 ± 0.13** | **0.33 ± 0.10** | **0.85 ± 0.17** | **0.44 ± 0.15** | **0.36 ± 0.12** | **0.19 ± 0.27** | **0.62 ± 0.16** |
| **wav2vec 2.0 feature extractor** | DT | 0.48 | 0.35 | 0.58 | 0.30 | 0.30 | -0.06 | 0.47 |
|  | KNN | 0.66 | 0.20 | 1.00 | 0.30 | 0.23 | 0.22 | 0.67 |
|  | LR | 0.58 | 0.00 | 1.00 | 0.00 | 0.00 | 0.00 | 0.75 |
|  | NB | 0.68 | 0.65 | 0.72 | 0.55 | 0.58 | 0.37 | 0.82 |
|  | RF | 0.58 | 0.35 | 0.77 | 0.30 | 0.30 | 0.11 | 0.72 |
|  | SVM | 0.58 | 0.00 | 1.00 | 0.00 | 0.00 | 0.00 | 0.38 |
|  | **mean ± std** | **0.59 ± 0.07** | **0.26 ± 0.25** | **0.84 ± 0.18** | **0.24 ± 0.21** | **0.24 ± 0.22** | **0.11 ± 0.16** | **0.63 ± 0.17** |
| **wav2vec 2.0 last hidden layer** | DT | 0.79 | 0.75 | 0.87 | 0.77 | 0.71 | 0.64 | 0.81 |
|  | KNN | 0.66 | 0.55 | 0.78 | 0.60 | 0.53 | 0.36 | 0.66 |
|  | LR | 0.58 | 0.00 | 1.00 | 0.00 | 0.00 | 0.00 | 0.38 |
|  | NB | 0.79 | 0.75 | 0.80 | 0.65 | 0.68 | 0.55 | 0.85 |
|  | RF | 0.79 | 0.55 | 0.95 | 0.67 | 0.58 | 0.53 | 0.68 |
|  | SVM | 0.58 | 0.00 | 1.00 | 0.00 | 0.00 | 0.00 | 0.43 |
|  | **mean ± std** | **0.70 ± 0.11** | **0.43 ± 0.35** | **0.90 ± 0.10** | **0.45 ± 0.35** | **0.42 ± 0.33** | **0.35 ± 0.28** | **0.64 ± 0.19** |
| **wav2vec 2.0 first transformer layer** | DT | 0.56 | 0.55 | 0.60 | 0.48 | 0.48 | 0.16 | 0.58 |
|  | KNN | 0.61 | 0.15 | 0.95 | 0.20 | 0.17 | 0.10 | 0.76 |
|  | LR | 0.73 | 0.65 | 0.80 | 0.62 | 0.60 | 0.47 | 0.95 |
|  | NB | 0.76 | 0.60 | 0.85 | 0.62 | 0.60 | 0.47 | 0.80 |
|  | RF | 0.53 | 0.30 | 0.75 | 0.28 | 0.27 | 0.06 | 0.63 |
|  | SVM | 0.76 | 0.65 | 0.85 | 0.67 | 0.63 | 0.52 | 0.90 |
|  | **mean ± std** | **0.66 ± 0.10** | **0.48 ± 0.21** | **0.80 ± 0.12** | **0.48 ± 0.19** | **0.46 ± 0.20** | **0.30 ± 0.21** | **0.77 ± 0.15** |

**Table D3.** Classifier performance metrics for dialogue from the English dataset, which included 21 HC and 15 PD samples in an imbalanced distribution. Results reported for the 30 PCA components.

|  | **classifier** | **accuracy** | **sens.** | **spec.** | **precision** | **F1** | **MCC** | **AUC** |
| --- | --- | --- | --- | --- | --- | --- | --- | --- |
| **wav2vec 1.0 feature extractor** | DT | 0.51 | 0.40 | 0.58 | 0.25 | 0.30 | -0.01 | 0.49 |
|  | KNN | 0.72 | 0.45 | 0.92 | 0.50 | 0.47 | 0.37 | 0.61 |
|  | LR | 0.59 | 0.00 | 1.00 | 0.00 | 0.00 | 0.00 | 0.82 |
|  | NB | 0.63 | 0.50 | 0.70 | 0.42 | 0.43 | 0.22 | 0.58 |
|  | RF | 0.51 | 0.10 | 0.77 | 0.10 | 0.10 | -0.14 | 0.43 |
|  | SVM | 0.59 | 0.00 | 1.00 | 0.00 | 0.00 | 0.00 | 0.62 |
|  | **mean ± std** | **0.59 ± 0.08** | **0.24 ± 0.23** | **0.83 ± 0.17** | **0.21 ± 0.21** | **0.22 ± 0.21** | **0.07 ± 0.18** | **0.59 ± 0.13** |
| **wav2vec 1.0 feature extractor + aggregator** | DT | 0.50 | 0.45 | 0.57 | 0.28 | 0.33 | 0.00 | 0.51 |
|  | KNN | 0.68 | 0.55 | 0.82 | 0.50 | 0.50 | 0.35 | 0.62 |
|  | LR | 0.72 | 0.50 | 0.92 | 0.50 | 0.48 | 0.42 | 0.79 |
|  | NB | 0.62 | 0.35 | 0.75 | 0.43 | 0.36 | 0.14 | 0.68 |
|  | RF | 0.53 | 0.10 | 0.80 | 0.20 | 0.13 | -0.08 | 0.55 |
|  | SVM | 0.72 | 0.50 | 0.92 | 0.50 | 0.48 | 0.42 | 0.69 |
|  | **mean ± std** | **0.63 ± 0.10** | **0.41 ± 0.17** | **0.79 ± 0.13** | **0.40 ± 0.13** | **0.38 ± 0.14** | **0.21 ± 0.22** | **0.64 ± 0.10** |
| **wav2vec 2.0 feature extractor** | DT | 0.78 | 0.80 | 0.78 | 0.65 | 0.70 | 0.58 | 0.79 |
|  | KNN | 0.66 | 0.40 | 0.87 | 0.45 | 0.40 | 0.27 | 0.68 |
|  | LR | 0.59 | 0.00 | 1.00 | 0.00 | 0.00 | 0.00 | 0.72 |
|  | NB | 0.50 | 0.25 | 0.67 | 0.22 | 0.21 | -0.10 | 0.42 |
|  | RF | 0.59 | 0.30 | 0.82 | 0.28 | 0.27 | 0.11 | 0.52 |
|  | SVM | 0.59 | 0.00 | 1.00 | 0.00 | 0.00 | 0.00 | 0.42 |
|  | **mean ± std** | **0.62 ± 0.09** | **0.29 ± 0.30** | **0.86 ± 0.13** | **0.27 ± 0.26** | **0.26 ± 0.26** | **0.14 ± 0.25** | **0.59 ± 0.16** |
| **wav2vec 2.0 last hidden layer** | DT | 0.54 | 0.40 | 0.57 | 0.33 | 0.36 | -0.04 | 0.48 |
|  | KNN | 0.44 | 0.10 | 0.67 | 0.13 | 0.11 | -0.24 | 0.38 |
|  | LR | 0.59 | 0.00 | 1.00 | 0.00 | 0.00 | 0.00 | 0.30 |
|  | NB | 0.63 | 0.35 | 0.80 | 0.42 | 0.36 | 0.17 | 0.59 |
|  | RF | 0.68 | 0.40 | 0.85 | 0.50 | 0.43 | 0.26 | 0.58 |
|  | SVM | 0.59 | 0.00 | 1.00 | 0.00 | 0.00 | 0.00 | 0.53 |
|  | **mean ± std** | **0.58 ± 0.08** | **0.21 ± 0.20** | **0.81 ± 0.18** | **0.23 ± 0.22** | **0.21 ± 0.20** | **0.02 ± 0.17** | **0.48 ± 0.11** |
| **wav2vec 2.0 first transformer layer** | DT | 0.53 | 0.60 | 0.52 | 0.43 | 0.47 | 0.12 | 0.56 |
|  | KNN | 0.68 | 0.55 | 0.82 | 0.53 | 0.51 | 0.37 | 0.66 |
|  | LR | 0.67 | 0.55 | 0.77 | 0.50 | 0.52 | 0.30 | 0.73 |
|  | NB | 0.36 | 0.15 | 0.47 | 0.12 | 0.13 | -0.41 | 0.37 |
|  | RF | 0.53 | 0.25 | 0.77 | 0.25 | 0.23 | 0.01 | 0.53 |
|  | SVM | 0.60 | 0.55 | 0.67 | 0.55 | 0.52 | 0.22 | 0.73 |
|  | **mean ± std** | **0.56 ± 0.12** | **0.44 ± 0.19** | **0.67 ± 0.14** | **0.40 ± 0.18** | **0.40 ± 0.17** | **0.10 ± 0.28** | **0.59 ± 0.14** |

**Table D4.** Classifier performance metrics for read text from the PC-GITA dataset, which included 50 HC and 50 PD samples in a balanced distribution. Results reported for the 30 PCA components.

|  | **classifier** | **accuracy** | **sens.** | **spec.** | **precision** | **F1** | **MCC** | **AUC** |
| --- | --- | --- | --- | --- | --- | --- | --- | --- |
| **wav2vec 1.0 feature extractor** | DT | 0.65 | 0.74 | 0.56 | 0.64 | 0.67 | 0.30 | 0.65 |
|  | KNN | 0.77 | 0.62 | 0.92 | 0.91 | 0.71 | 0.58 | 0.82 |
|  | LR | 0.69 | 0.58 | 0.80 | 0.76 | 0.64 | 0.40 | 0.75 |
|  | NB | 0.67 | 0.66 | 0.68 | 0.71 | 0.67 | 0.36 | 0.77 |
|  | RF | 0.74 | 0.74 | 0.74 | 0.78 | 0.75 | 0.50 | 0.80 |
|  | SVM | 0.66 | 0.50 | 0.82 | 0.75 | 0.59 | 0.35 | 0.34 |
|  | **mean ± std** | **0.70 ± 0.05** | **0.64 ± 0.09** | **0.75 ± 0.12** | **0.76 ± 0.09** | **0.67 ± 0.05** | **0.41 ± 0.11** | **0.69 ± 0.18** |
| **wav2vec 1.0 feature extractor + aggregator** | DT | 0.63 | 0.70 | 0.56 | 0.62 | 0.65 | 0.27 | 0.63 |
|  | KNN | 0.77 | 0.62 | 0.92 | 0.87 | 0.71 | 0.57 | 0.83 |
|  | LR | 0.75 | 0.68 | 0.82 | 0.80 | 0.71 | 0.52 | 0.87 |
|  | NB | 0.68 | 0.68 | 0.68 | 0.68 | 0.67 | 0.37 | 0.74 |
|  | RF | 0.76 | 0.80 | 0.72 | 0.74 | 0.76 | 0.54 | 0.80 |
|  | SVM | 0.81 | 0.76 | 0.86 | 0.86 | 0.78 | 0.65 | 0.89 |
|  | **mean ± std** | **0.73 ± 0.07** | **0.71 ± 0.06** | **0.76 ± 0.13** | **0.76 ± 0.10** | **0.71 ± 0.05** | **0.49 ± 0.14** | **0.79 ± 0.10** |
| **wav2vec 2.0 feature extractor** | DT | 0.64 | 0.58 | 0.70 | 0.65 | 0.60 | 0.28 | 0.64 |
|  | KNN | 0.75 | 0.66 | 0.84 | 0.83 | 0.71 | 0.53 | 0.82 |
|  | LR | 0.68 | 0.76 | 0.60 | 0.70 | 0.71 | 0.37 | 0.77 |
|  | NB | 0.70 | 0.68 | 0.72 | 0.75 | 0.70 | 0.42 | 0.76 |
|  | RF | 0.72 | 0.68 | 0.76 | 0.78 | 0.70 | 0.46 | 0.80 |
|  | SVM | 0.69 | 0.78 | 0.60 | 0.71 | 0.73 | 0.39 | 0.76 |
|  | **mean ± std** | **0.70 ± 0.04** | **0.69 ± 0.07** | **0.70 ± 0.09** | **0.74 ± 0.06** | **0.69 ± 0.04** | **0.41 ± 0.08** | **0.76 ± 0.06** |
| **wav2vec 2.0 last hidden layer** | DT | 0.74 | 0.72 | 0.76 | 0.78 | 0.72 | 0.51 | 0.74 |
|  | KNN | 0.56 | 0.48 | 0.64 | 0.58 | 0.51 | 0.13 | 0.63 |
|  | LR | 0.41 | 0.34 | 0.48 | 0.41 | 0.36 | -0.19 | 0.38 |
|  | NB | 0.71 | 0.66 | 0.76 | 0.75 | 0.69 | 0.43 | 0.80 |
|  | RF | 0.77 | 0.80 | 0.74 | 0.78 | 0.76 | 0.58 | 0.88 |
|  | SVM | 0.45 | 0.18 | 0.72 | 0.33 | 0.21 | -0.12 | 0.56 |
|  | **mean ± std** | **0.61 ± 0.16** | **0.53 ± 0.24** | **0.68 ± 0.11** | **0.61 ± 0.20** | **0.54 ± 0.22** | **0.22 ± 0.33** | **0.66 ± 0.18** |
| **wav2vec 2.0 first transformer layer** | DT | 0.65 | 0.64 | 0.66 | 0.67 | 0.64 | 0.32 | 0.65 |
|  | KNN | 0.76 | 0.68 | 0.84 | 0.84 | 0.73 | 0.55 | 0.78 |
|  | LR | 0.81 | 0.78 | 0.84 | 0.86 | 0.81 | 0.64 | 0.83 |
|  | NB | 0.74 | 0.74 | 0.74 | 0.77 | 0.74 | 0.50 | 0.84 |
|  | RF | 0.75 | 0.78 | 0.72 | 0.77 | 0.76 | 0.51 | 0.77 |
|  | SVM | 0.79 | 0.80 | 0.78 | 0.82 | 0.79 | 0.60 | 0.80 |
|  | **mean ± std** | **0.75 ± 0.06** | **0.74 ± 0.06** | **0.76 ± 0.07** | **0.79 ± 0.07** | **0.74 ± 0.06** | **0.52 ± 0.11** | **0.78 ± 0.07** |

**Table D5.** Classifier performance metrics for monologue from the PC-GITA dataset, which included 50 HC and 50 PD samples in a balanced distribution. Results reported for the 30 PCA components.

|  | **classifier** | **accuracy** | **sens.** | **spec.** | **precision** | **F1** | **MCC** | **AUC** |
| --- | --- | --- | --- | --- | --- | --- | --- | --- |
| **wav2vec 1.0 feature extractor** | DT | 0.65 | 0.64 | 0.66 | 0.66 | 0.64 | 0.32 | 0.65 |
|  | KNN | 0.75 | 0.68 | 0.82 | 0.74 | 0.70 | 0.50 | 0.83 |
|  | LR | 0.69 | 0.62 | 0.76 | 0.73 | 0.65 | 0.39 | 0.80 |
|  | NB | 0.81 | 0.84 | 0.78 | 0.80 | 0.82 | 0.62 | 0.86 |
|  | RF | 0.77 | 0.72 | 0.82 | 0.83 | 0.74 | 0.57 | 0.86 |
|  | SVM | 0.65 | 0.52 | 0.78 | 0.73 | 0.58 | 0.33 | 0.39 |
|  | **mean ± std** | **0.72 ± 0.07** | **0.67 ± 0.11** | **0.77 ± 0.06** | **0.75 ± 0.06** | **0.69 ± 0.08** | **0.45 ± 0.13** | **0.73 ± 0.19** |
| **wav2vec 1.0 feature extractor + aggregator** | DT | 0.67 | 0.68 | 0.66 | 0.68 | 0.66 | 0.35 | 0.67 |
|  | KNN | 0.76 | 0.72 | 0.80 | 0.80 | 0.75 | 0.54 | 0.80 |
|  | LR | 0.77 | 0.74 | 0.80 | 0.81 | 0.76 | 0.56 | 0.87 |
|  | NB | 0.73 | 0.70 | 0.76 | 0.75 | 0.71 | 0.48 | 0.80 |
|  | RF | 0.69 | 0.68 | 0.70 | 0.70 | 0.67 | 0.41 | 0.79 |
|  | SVM | 0.79 | 0.74 | 0.84 | 0.84 | 0.77 | 0.61 | 0.90 |
|  | **mean ± std** | **0.74 ± 0.05** | **0.71 ± 0.03** | **0.76 ± 0.07** | **0.76 ± 0.06** | **0.72 ± 0.05** | **0.49 ± 0.10** | **0.81 ± 0.08** |
| **wav2vec 2.0 feature extractor** | DT | 0.66 | 0.62 | 0.70 | 0.67 | 0.64 | 0.33 | 0.66 |
|  | KNN | 0.78 | 0.74 | 0.82 | 0.83 | 0.76 | 0.58 | 0.83 |
|  | LR | 0.69 | 0.74 | 0.64 | 0.68 | 0.70 | 0.40 | 0.78 |
|  | NB | 0.73 | 0.70 | 0.76 | 0.76 | 0.72 | 0.47 | 0.80 |
|  | RF | 0.74 | 0.76 | 0.72 | 0.75 | 0.75 | 0.49 | 0.81 |
|  | SVM | 0.69 | 0.80 | 0.58 | 0.67 | 0.72 | 0.41 | 0.79 |
|  | **mean ± std** | **0.72 ± 0.04** | **0.73 ± 0.06** | **0.70 ± 0.09** | **0.73 ± 0.06** | **0.71 ± 0.04** | **0.45 ± 0.09** | **0.78 ± 0.06** |
| **wav2vec 2.0 last hidden layer** | DT | 0.61 | 0.64 | 0.58 | 0.64 | 0.63 | 0.23 | 0.61 |
|  | KNN | 0.61 | 0.54 | 0.68 | 0.65 | 0.56 | 0.24 | 0.66 |
|  | LR | 0.56 | 0.52 | 0.60 | 0.59 | 0.52 | 0.14 | 0.63 |
|  | NB | 0.76 | 0.78 | 0.74 | 0.76 | 0.75 | 0.55 | 0.89 |
|  | RF | 0.74 | 0.68 | 0.80 | 0.80 | 0.72 | 0.50 | 0.80 |
|  | SVM | 0.53 | 0.08 | 0.98 | 0.35 | 0.13 | 0.10 | 0.39 |
|  | **mean ± std** | **0.64 ± 0.09** | **0.54 ± 0.24** | **0.73 ± 0.15** | **0.63 ± 0.16** | **0.55 ± 0.23** | **0.29 ± 0.19** | **0.66 ± 0.17** |
| **wav2vec 2.0 first transformer layer** | DT | 0.54 | 0.56 | 0.52 | 0.54 | 0.54 | 0.07 | 0.54 |
|  | KNN | 0.75 | 0.72 | 0.78 | 0.78 | 0.74 | 0.52 | 0.83 |
|  | LR | 0.80 | 0.80 | 0.80 | 0.82 | 0.80 | 0.62 | 0.90 |
|  | NB | 0.75 | 0.82 | 0.68 | 0.72 | 0.77 | 0.51 | 0.82 |
|  | RF | 0.74 | 0.76 | 0.72 | 0.74 | 0.75 | 0.48 | 0.79 |
|  | SVM | 0.77 | 0.78 | 0.76 | 0.81 | 0.77 | 0.57 | 0.88 |
|  | **mean ± std** | **0.73 ± 0.09** | **0.74 ± 0.09** | **0.71 ± 0.10** | **0.74 ± 0.10** | **0.73 ± 0.09** | **0.46 ± 0.20** | **0.79 ± 0.13** |

**Table D6.** Classifier performance metrics for vowel dataset from the PC-GITA dataset, which included 50 HC and 50 PD samples in a balanced distribution. Results reported for the 30 PCA components.

|  | **classifier** | **accuracy** | **sens.** | **spec.** | **precision** | **F1** | **MCC** | **AUC** |
| --- | --- | --- | --- | --- | --- | --- | --- | --- |
| **wav2vec 1.0 feature extractor** | DT | 0.59 | 0.64 | 0.54 | 0.60 | 0.61 | 0.18 | 0.59 |
|  | KNN | 0.62 | 0.64 | 0.60 | 0.63 | 0.62 | 0.25 | 0.65 |
|  | LR | 0.65 | 0.64 | 0.66 | 0.67 | 0.64 | 0.32 | 0.71 |
|  | NB | 0.63 | 0.68 | 0.58 | 0.63 | 0.65 | 0.26 | 0.66 |
|  | RF | 0.64 | 0.72 | 0.56 | 0.63 | 0.66 | 0.29 | 0.69 |
|  | SVM | 0.61 | 0.44 | 0.78 | 0.72 | 0.51 | 0.26 | 0.73 |
|  | **mean ± std** | **0.62 ± 0.02** | **0.63 ± 0.10** | **0.62 ± 0.09** | **0.65 ± 0.05** | **0.61 ± 0.05** | **0.26 ± 0.05** | **0.67 ± 0.05** |
| **wav2vec 1.0 feature extractor + aggregator** | DT | 0.44 | 0.42 | 0.46 | 0.40 | 0.40 | -0.13 | 0.44 |
|  | KNN | 0.53 | 0.60 | 0.46 | 0.53 | 0.55 | 0.04 | 0.57 |
|  | LR | 0.58 | 0.54 | 0.62 | 0.62 | 0.55 | 0.17 | 0.68 |
|  | NB | 0.63 | 0.54 | 0.72 | 0.65 | 0.57 | 0.27 | 0.67 |
|  | RF | 0.56 | 0.52 | 0.60 | 0.58 | 0.53 | 0.13 | 0.59 |
|  | SVM | 0.55 | 0.50 | 0.60 | 0.58 | 0.52 | 0.11 | 0.69 |
|  | **mean ± std** | **0.55 ± 0.06** | **0.52 ± 0.06** | **0.58 ± 0.10** | **0.56 ± 0.09** | **0.52 ± 0.06** | **0.10 ± 0.13** | **0.61 ± 0.10** |
| **wav2vec 2.0 feature extractor** | DT | 0.53 | 0.60 | 0.46 | 0.53 | 0.55 | 0.07 | 0.53 |
|  | KNN | 0.74 | 0.76 | 0.72 | 0.76 | 0.75 | 0.49 | 0.80 |
|  | LR | 0.71 | 0.76 | 0.66 | 0.71 | 0.73 | 0.43 | 0.83 |
|  | NB | 0.76 | 0.76 | 0.76 | 0.78 | 0.76 | 0.53 | 0.80 |
|  | RF | 0.69 | 0.76 | 0.62 | 0.69 | 0.71 | 0.39 | 0.81 |
|  | SVM | 0.73 | 0.72 | 0.74 | 0.77 | 0.73 | 0.47 | 0.80 |
|  | **mean ± std** | **0.69 ± 0.08** | **0.73 ± 0.06** | **0.66 ± 0.11** | **0.71 ± 0.09** | **0.71 ± 0.08** | **0.40 ± 0.17** | **0.76 ± 0.11** |
| **wav2vec 2.0 last hidden layer** | DT | 0.63 | 0.58 | 0.68 | 0.62 | 0.59 | 0.26 | 0.63 |
|  | KNN | 0.68 | 0.72 | 0.64 | 0.71 | 0.69 | 0.38 | 0.67 |
|  | LR | 0.59 | 0.48 | 0.70 | 0.61 | 0.53 | 0.19 | 0.64 |
|  | NB | 0.63 | 0.62 | 0.64 | 0.62 | 0.61 | 0.27 | 0.67 |
|  | RF | 0.69 | 0.70 | 0.68 | 0.70 | 0.69 | 0.39 | 0.78 |
|  | SVM | 0.56 | 0.36 | 0.76 | 0.62 | 0.43 | 0.14 | 0.42 |
|  | **mean ± std** | **0.63 ± 0.05** | **0.58 ± 0.14** | **0.68 ± 0.04** | **0.65 ± 0.04** | **0.59 ± 0.10** | **0.27 ± 0.10** | **0.63 ± 0.12** |
| **wav2vec 2.0 first transformer layer** | DT | 0.62 | 0.74 | 0.50 | 0.60 | 0.65 | 0.28 | 0.62 |
|  | KNN | 0.73 | 0.76 | 0.70 | 0.72 | 0.74 | 0.47 | 0.79 |
|  | LR | 0.70 | 0.72 | 0.68 | 0.70 | 0.70 | 0.41 | 0.78 |
|  | NB | 0.72 | 0.74 | 0.70 | 0.72 | 0.72 | 0.45 | 0.72 |
|  | RF | 0.68 | 0.70 | 0.66 | 0.68 | 0.68 | 0.36 | 0.76 |
|  | SVM | 0.74 | 0.78 | 0.70 | 0.74 | 0.75 | 0.50 | 0.78 |
|  | **mean ± std** | **0.70 ± 0.04** | **0.74 ± 0.03** | **0.66 ± 0.08** | **0.69 ± 0.05** | **0.71 ± 0.04** | **0.41 ± 0.08** | **0.74 ± 0.07** |

**Table D7.** Classifier performance metrics for vowel dataset from the U.S.-based dataset, which included 41 HC and 40 PD samples in a balanced distribution. Results reported for the 30 PCA components.

|  | **classifier** | **accuracy** | **sens.** | **spec.** | **precision** | **F1** | **MCC** | **AUC** |
| --- | --- | --- | --- | --- | --- | --- | --- | --- |
| **wav2vec 1.0 feature extractor** | DT | 0.52 | 0.60 | 0.44 | 0.52 | 0.54 | 0.06 | 0.52 |
|  | KNN | 0.54 | 0.70 | 0.40 | 0.58 | 0.59 | 0.12 | 0.61 |
|  | LR | 0.63 | 0.65 | 0.62 | 0.64 | 0.63 | 0.28 | 0.68 |
|  | NB | 0.67 | 0.63 | 0.71 | 0.71 | 0.64 | 0.35 | 0.68 |
|  | RF | 0.65 | 0.65 | 0.65 | 0.68 | 0.65 | 0.32 | 0.69 |
|  | SVM | 0.63 | 0.63 | 0.64 | 0.66 | 0.61 | 0.29 | 0.37 |
|  | **mean ± std** | **0.61 ± 0.06** | **0.64 ± 0.03** | **0.57 ± 0.13** | **0.63 ± 0.07** | **0.61 ± 0.04** | **0.24 ± 0.12** | **0.59 ± 0.13** |
| **wav2vec 1.0 feature extractor + aggregator** | DT | 0.50 | 0.55 | 0.43 | 0.48 | 0.50 | -0.04 | 0.49 |
|  | KNN | 0.69 | 0.70 | 0.68 | 0.74 | 0.69 | 0.41 | 0.72 |
|  | LR | 0.59 | 0.65 | 0.54 | 0.63 | 0.61 | 0.20 | 0.65 |
|  | NB | 0.53 | 0.60 | 0.46 | 0.53 | 0.56 | 0.05 | 0.50 |
|  | RF | 0.61 | 0.63 | 0.59 | 0.55 | 0.58 | 0.20 | 0.65 |
|  | SVM | 0.64 | 0.68 | 0.61 | 0.65 | 0.65 | 0.29 | 0.71 |
|  | **mean ± std** | **0.59 ± 0.07** | **0.63 ± 0.05** | **0.55 ± 0.09** | **0.60 ± 0.09** | **0.60 ± 0.07** | **0.19 ± 0.16** | **0.62 ± 0.10** |
| **wav2vec 2.0 feature extractor** | DT | 0.64 | 0.65 | 0.63 | 0.70 | 0.64 | 0.31 | 0.64 |
|  | KNN | 0.65 | 0.73 | 0.58 | 0.68 | 0.66 | 0.34 | 0.68 |
|  | LR | 0.71 | 0.75 | 0.68 | 0.72 | 0.72 | 0.45 | 0.80 |
|  | NB | 0.62 | 0.55 | 0.68 | 0.60 | 0.54 | 0.25 | 0.68 |
|  | RF | 0.67 | 0.68 | 0.66 | 0.68 | 0.65 | 0.36 | 0.74 |
|  | SVM | 0.71 | 0.75 | 0.68 | 0.72 | 0.72 | 0.45 | 0.83 |
|  | **mean ± std** | **0.67 ± 0.04** | **0.68 ± 0.08** | **0.65 ± 0.04** | **0.68 ± 0.04** | **0.65 ± 0.07** | **0.36 ± 0.08** | **0.73 ± 0.07** |
| **wav2vec 2.0 last hidden layer** | DT | 0.63 | 0.68 | 0.58 | 0.67 | 0.65 | 0.28 | 0.63 |
|  | KNN | 0.64 | 0.75 | 0.53 | 0.62 | 0.67 | 0.29 | 0.72 |
|  | LR | 0.62 | 0.40 | 0.83 | 0.59 | 0.46 | 0.24 | 0.72 |
|  | NB | 0.62 | 0.58 | 0.66 | 0.64 | 0.59 | 0.25 | 0.68 |
|  | RF | 0.59 | 0.70 | 0.48 | 0.59 | 0.63 | 0.17 | 0.69 |
|  | SVM | 0.51 | 0.00 | 1.00 | 0.00 | 0.00 | 0.00 | 0.29 |
|  | **mean ± std** | **0.60 ± 0.05** | **0.52 ± 0.28** | **0.68 ± 0.20** | **0.52 ± 0.26** | **0.50 ± 0.26** | **0.20 ± 0.11** | **0.62 ± 0.17** |
| **wav2vec 2.0 first transformer layer** | DT | 0.62 | 0.63 | 0.61 | 0.64 | 0.62 | 0.25 | 0.62 |
|  | KNN | 0.67 | 0.68 | 0.66 | 0.71 | 0.65 | 0.37 | 0.71 |
|  | LR | 0.64 | 0.63 | 0.66 | 0.65 | 0.62 | 0.30 | 0.69 |
|  | NB | 0.60 | 0.55 | 0.67 | 0.61 | 0.55 | 0.23 | 0.65 |
|  | RF | 0.65 | 0.65 | 0.66 | 0.65 | 0.63 | 0.32 | 0.69 |
|  | SVM | 0.60 | 0.55 | 0.66 | 0.63 | 0.55 | 0.23 | 0.69 |
|  | **mean ± std** | **0.63 ± 0.03** | **0.61 ± 0.05** | **0.65 ± 0.02** | **0.65 ± 0.04** | **0.60 ± 0.04** | **0.28 ± 0.06** | **0.68 ± 0.03** |
